# A Loss of Function Screen of Identified Genome-Wide Association Study Loci Reveals New Genes Controlling Hematopoiesis

**DOI:** 10.1371/journal.pgen.1004450

**Published:** 2014-07-10

**Authors:** Ewa Bielczyk-Maczyńska, Jovana Serbanovic-Canic, Lauren Ferreira, Nicole Soranzo, Derek L. Stemple, Willem H. Ouwehand, Ana Cvejic

**Affiliations:** 1 Department of Haematology, University of Cambridge, Cambridge, United Kingdom; 2 Wellcome Trust Sanger Institute, Wellcome Trust Genome Campus, Hinxton, Cambridge, United Kingdom; 3 NHS Blood and Transplant, Cambridge, United Kingdom; 4 MRC Centre for Developmental and Biomedical Genetics, University of Sheffield, Sheffield, United Kingdom; 5 Department of Cardiovascular Sciences, University of Sheffield, Sheffield, United Kingdom; Children's Hospital, United States of America

## Abstract

The formation of mature cells by blood stem cells is very well understood at the cellular level and we know many of the key transcription factors that control fate decisions. However, many upstream signalling and downstream effector processes are only partially understood. Genome wide association studies (GWAS) have been particularly useful in providing new directions to dissect these pathways. A GWAS meta-analysis identified 68 genetic loci controlling platelet size and number. Only a quarter of those genes, however, are known regulators of hematopoiesis. To determine function of the remaining genes we performed a medium-throughput genetic screen in zebrafish using antisense morpholino oligonucleotides (MOs) to knock down protein expression, followed by histological analysis of selected genes using a wide panel of different hematopoietic markers. The information generated by the initial knockdown was used to profile phenotypes and to position candidate genes hierarchically in hematopoiesis. Further analysis of *brd3a* revealed its essential role in differentiation but not maintenance and survival of thrombocytes. Using the from-GWAS-to-function strategy we have not only identified a series of genes that represent novel regulators of thrombopoiesis and hematopoiesis, but this work also represents, to our knowledge, the first example of a functional genetic screening strategy that is a critical step toward obtaining biologically relevant functional data from GWA study for blood cell traits.

## Author Summary

In this manuscript we report on a follow-up study of the GWAS loci associated with the platelet size and number. A GWAS meta-analysis identified 68 genetic loci controlling platelet size and number. Only a quarter of those genes, however, are known regulators of hematopoiesis. To determine function of the remaining genes we performed a medium-throughput genetic screen in zebrafish using morpholinos (MOs) to knock down selected candidate genes. Here, we report on two major findings. First we identified 15 genes (corresponding to 12 human genes) required for distinct stages of specification or differentiation of HSCs in zebrafish. A detailed review of databases and literature revealed limited knowledge about the functional role of *Satb1*, *Rcor1* and *Brd3* in hematopoiesis and for the remaining nine genes our work represents the first study on their putative role in hematopoiesis. And secondly, we demonstrate that *brd3a* is critical for establishing, but not maintaining thrombopoietic compartment. Importantly, our study introduces zebrafish as a model system for functional follow-up of GWAS loci and generates a valuable resource for prioritization of platelet size and number associated genes for future in-depth mechanistic analyses. Following this route of investigation new regulatory molecules of hematopoiesis will be added to critical pathways.

## Introduction

Erythrocytes and platelets (thrombocytes in zebrafish) are the most abundant cells in blood. In an individual, the number and volume of both erythrocytes and platelets are highly heritable and tightly regulated within narrow ranges, but there is a wide variation of these parameters in the population [1], [2]. The 80% heritability of blood cell indices provided the foundation for our recently completed GWAS meta-analysis in ∼68,000 healthy individuals for both cell types. We identified 68 genetic loci that control the mass (volume x count) of platelets [3] and another 75 for red cell indices [4]. About a quarter of the genes proximal to the platelet GWAS association single nucleotide polymorphisms (SNPs) encode well studied and generally pivotal regulators of hematopoiesis but the function of the remaining ones is unknown, demonstrating the power of GWAS to identify novel regulators of hematopoiesis. We have recently reported functional validation of six genes in which the sentinel SNP was localized within a gene and silenced *ak3*, *rnf145*, *arhgef3*, *tpm1*, *jmjd1c* and *ehd3* in zebrafish by MO injections. Profound effects on thrombopoiesis were observed for all but *ehd3* [3]. Furthermore, our detailed studies of the *arhgef3* gene, which encodes one of the ∼70 Rho guanine nucleotide exchange factors, showed its important role in iron uptake and transferrin receptor internalisation in erythrocytes [5]. Based on these preliminary data, we hypothesized that the majority of genes identified in our recent genomics efforts are important and rate-limiting regulators of hematopoiesis and therefore worthwhile of further investigation.

The zebrafish model has distinct advantages over other animal models for screening large numbers of genes. Zebrafish development occurs rapidly over the course of a few days with thrombocytes, erythroid- and myeloid- blood cells being fully formed and functional by 3 days post fertilisation (dpf). External fertilisation and transparency of zebrafish embryos allow easy visualisation of early blood-related phenotypes giving them the advantage over mice, where development occurs *in utero*. Importantly, transcriptional mechanisms and signalling pathways in hematopoiesis are well conserved between zebrafish and mammals [6].

Herein, we performed a MO injection screen of 15 genes identified in GWAS for platelet size and number to uncover novel pathways essential in thrombopoiesis and hematopoiesis in general. From this screen, we identified 12 new genes required for normal hematopoiesis and ordered them on a hematopoietic lineage tree based on their presumed function during hematopoiesis. Further analysis of the hematopoietic lineage tree revealed a distinct pattern of gene distribution suggesting two main gene clusters. One cluster of genes appears to work at the level of HSCs affecting all derived blood cell types and the second cluster appears to be limited to controlling the specification of the thrombocyte-erythroid progenitors. Additionally, we show that one of the novel candidate genes, *brd3a*, is essential in differentiation of thrombocytes from HSCs but is dispensable for their maintenance.

## Results

### Gene selection

To interrogate the large number of novel hematopoietic genes identified in the GWAS for platelet size and number we developed an *in vivo* functional genomics screen in zebrafish (Figure S1). The first step was selection of the most suitable candidate genes and initially we selected a single gene, closest to the sentinel SNP, from each GWAS locus [3] (Table S1). Distance from the nearest gene was calculated as the absolute distance between SNP and transcription start site of the gene or 3′ end of last exon [3] (Table S1). We further eliminated genes with a known function in hematopoiesis and identified a putative zebrafish ortholog, with over 38% identity, on the protein level, with its human counterpart for 40 genes. Finally, we excluded all genes that would require the use of more than two MOs, resulting in a list of 33 genes. Nearly 80% of these genes had a sentinel SNPs localized within 10 kb. In the last step, we selected 19 genes of which 16 had a sentinel SNP within 10 kb and three genes had a sentinel SNP >10 kb. Five of the selected genes were duplicated in zebrafish, resulting in a total of 24 genes to be further investigated aiming to define their function in blood cell formation by a MO knockdown approach in zebrafish.

We designed splice-blocking MOs for each gene and validated their efficacy with RT-PCR and sequencing. Of the 24 MOs tested five MOs had no effect on the target RNA and these MOs were excluded from further analysis (Figure S2). We then assayed the remaining 19 functional MOs for their effect on overall development, morphology and hematopoiesis during the first 72 hours post fertilisation (hpf) and selected the optimal dose of MO to be injected (Figure S3). Of note, based on the information available at the ZFIN at the time and our *in situ* hybridization data (Figure S4) none of the selected genes had hematopoietic specific gene expression. For all genes, except *grtp1a*, the optimal dose of MO was selected that resulted in a specific phenotype but without gross lethality or defects in body shape or size, vasculature, heart and circulation. We found that *grtp1a* MO injected embryos died by 15 hpf even when injected with 0.8 ng of MO, thus we excluded *grtp1a* from further analysis.

### A reverse genetic screen identified genes essential in hematopoiesis

Although morphological examination can detect defects with great sensitivity, some specific defects in hematopoiesis can be missed, and as a result information obtained from the initial analysis might be limited. Thus, we carried out a second level of analysis by performing *in situ* hybridisation with several hematopoietic markers, specifically, *c-myb*, *ae1 globin*, *mpeg* and *rag1*. These were complemented with the use of the *Tg(cd41:EGFP)* line and two histochemical stains, o-Dianisidine and Sudan black. The hematopoietic markers used for phenotyping were carefully chosen to distinguish between early and late stages of hematopoiesis as well as thrombocytes, erythrocytes, neutrophils, macrophages and lymphocytes (Figures S5).

**Figure 1:**
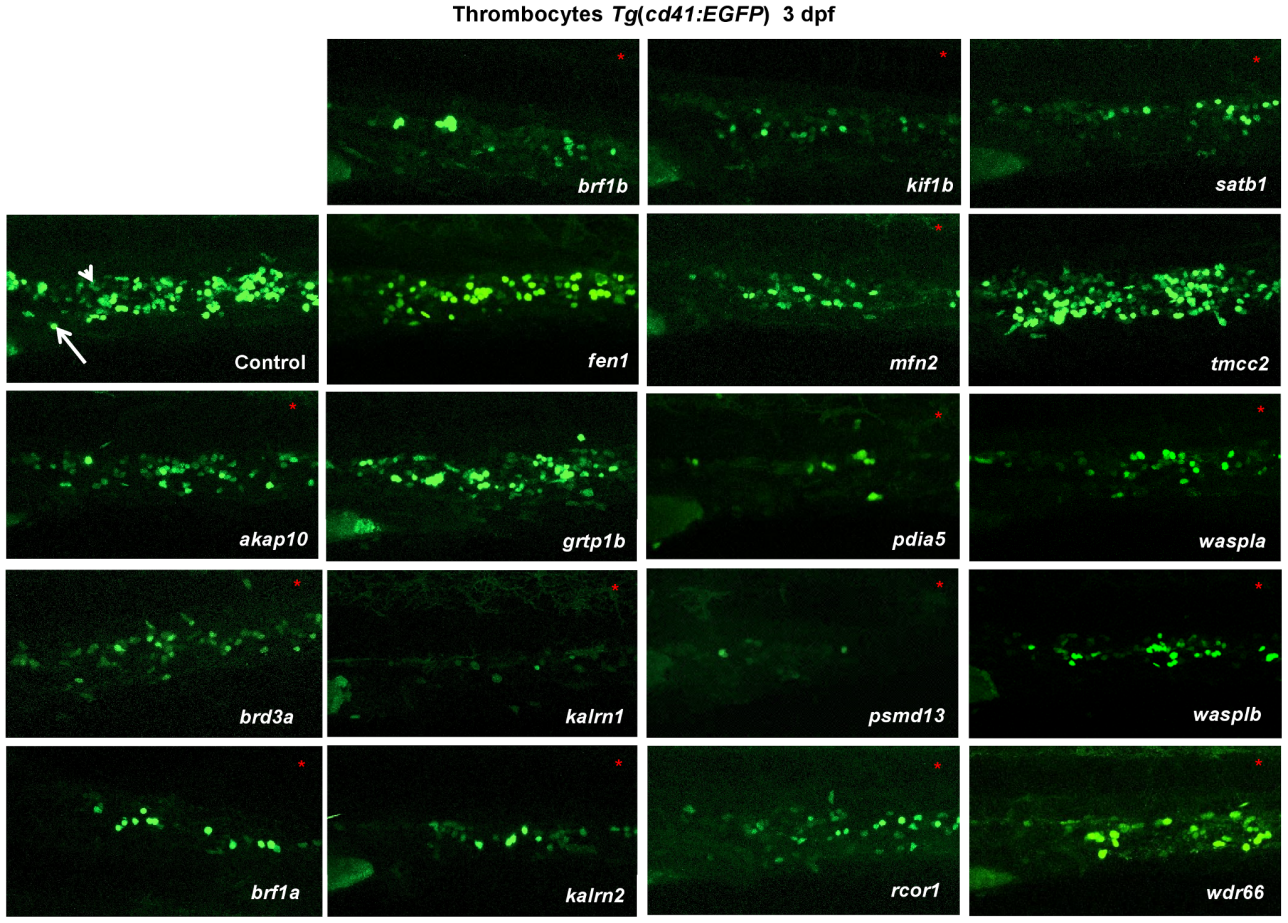
*In vivo* morpholino screen in zebrafish identifies 15 new regulators of thrombopoiesis. MOs were injected into one-cell stage transgenic *Tg(cd41:EGFP)* zebrafish embryos and assayed for their effect on the number of thrombocytes (*cd41^high^*) at 3 dpf. Representative confocal images were taken of the CHT. For *akap10*, *brd3a*, *brf1b*, *kalrn 1*, *kalrn 2*, *kif1b*, *mfn2*, *pdia5*, *psmd13* and *satb1* a severe decrease in the number of *cd41^high^* positive cells was observed. *brf1a*, *rcor1*, *waspla*, *wasplb* and *wdr66* depletion resulted in a mild phenotype, and *fen1*, *grtp1b* and *tmcc2* MO injected embryos showed no phenotype. All embryos are oriented with anterior to the left and dorsal to the top. White arrow – thrombocytes; white arrowhead – HSCs.

We initiated screening by taking advantage of the *Tg(cd41:EGFP)* reporter line, which labels thrombocytes, to identify genes in zebrafish that, when disrupted, affect thrombocyte number. We found that knock down of 15 of the 18 genes resulted in 30–95% reduction in thrombocyte number (Figure 1, Figure S6). Importantly, where the functional second MO was available, the observed phenotype was comparable to the one observed with the first MO (Figure S7, S8, Table S2). Furthermore, concurrent knock down of *p53* with gene specific MOs did not attenuate the thrombocyte phenotype induced by gene specific MOs, confirming that the observed decrease in the number of thrombocytes was not induced by *p53* mediated off target effects of MO injection (Figure S9). For one of the “phenotypic” genes, *brf1b*, we have obtained the mutant through ZMP. To test if *brf1b* mutants have a decreased number of thrombocytes, the offspring were subjected to the “clotting time assay” at 5 dpf [7]. Clotting time in *brf1b* mutant larvae was significantly longer than in the wild type fish, suggesting a defect in thrombopoiesis (Figure S10 A). To further confirm the specificity of the phenotype observed in MO injected embryos and in the absence of available mutants we performed the rescue of *rcor1* MO injected embryos using full-length zebrafish *rcor1* RNA (Figure S10 B, C). We focused our further analysis on the 15 “phenotypic” genes and their paralogs.

**Figure 2:**
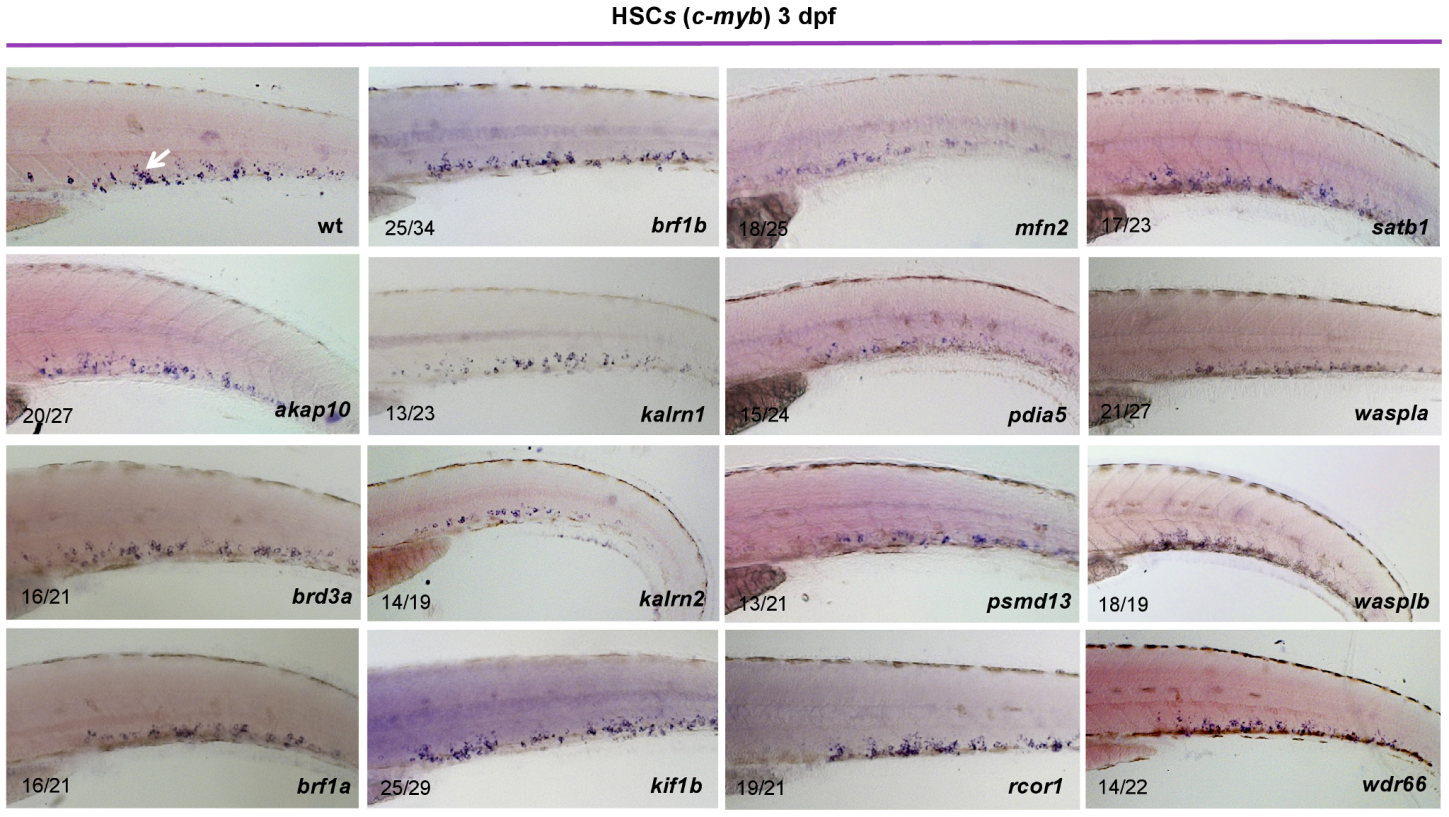
Characterization of HSCs in candidate gene depleted embryos. To assess the stage at which hematopoiesis of each MO injected embryo was defective, we performed whole mount *in situ* hybridization using a *c-myb* probe at 3 dpf. Although more than half of MOs had no effect on the number of HSCs, depletion of *rcor1* resulted in increased numbers of HSCs and depletion of *kalrn1*, *kalrn2*, *mfn2*, *pdia5*, *psmd13* and *wasplb* resulted in decreased numbers of HSCs in CHT at 3 dpf. Representative images of CHT region are shown. All embryos are oriented with anterior to the left and dorsal to the top.

To maintain the population of differentiated blood cells within normal ranges, HSCs need to continuously maintain the balance between self-renewal and differentiation. We reasoned that decreased number of thrombocytes in MO injected embryos could be the result of either reduced numbers of HSCs or altered HSC differentiation. To assess which stage of hematopoiesis was affected in each MO injected embryo, we performed *in situ* hybridization at 3 dpf and looked for alterations in expression of definitive hematopoiesis marker *c-myb* (Figure 2). Although more than half of the MOs tested had no effect on the number of HSCs, depletion of *rcor1* resulted in an increased number of HSCs and depletion of *kalrn (1 and 2)*, *mfn2*, *pdia5*, *psmd13* and *wasplb* resulted in decreased numbers of HSCs in caudal hematopoietic tissue (CHT) at 3 dpf. This reduction in the number of HSCs was not evident at 30 hpf in *kalrn1*, *mfn2*, *pdia5*, *psmd13* and *wasplb* MO injected embryos (Figure S11) suggesting that the number of HSCs at 3 dpf was most probably adversely affected by either their homing to or survival/proliferation in CHT. However, *kalrn2 and rcor1* depleted embryos had a marked decrease in the number of HSCs at 30 hpf, implying the important role of these genes in specification of HSCs in the aorta-gonad-mesonephros (AGM) (Figure S11). Importantly, analysis of vascular development by injecting candidate gene MOs into *Tg(fli1:EGFP)* embryos, which express EGFP in endothelial cells, demonstrated no major abnormalities in vascular morphogenesis or remodeling that would preclude circulation, indicating that the hematopoietic defects were not secondary to a vascular phenotype (Figure S12). Thus, our screen effectively defined a set of nine genes required for differentiation of HSCs to thrombocytes and possibly other blood lineages.

Hematopoiesis is often depicted by a hierarchical differentiation tree, with HSCs at the root and the mature blood cells as the branches. One of the intermediate cellular states is the common myeloid progenitor (CMP), which can proliferate and differentiate into megakaryocyte-erythrocyte progenitors (MEP) and granulocyte-monocyte (GM) progenitors, which further give rise to megakaryocytes, erythrocytes, granulocytes, monocytes and other cell types. To investigate lineage-specific effects of the candidate genes, we assessed the status of definitive erythropoiesis in MO injected embryos at 4 dpf. As α*e1-globin* RNA was reported to be expressed in definitive erythrocytes at 4 dpf [8], a detailed analysis of the expression pattern of α*e1-globin* transcript was exploited to reveal the initiation of definitive erythropoiesis after gene silencing. Profound effects on definitive erythropoiesis were observed for all but *brd3a*, *brf1b*, *waspla* and *wdr66* (Figure S13). However, silencing of *brf1a* and *wasplb* resulted in diminished definitive erythropoiesis, reflecting functional divergence of duplicated genes (*brf1* and *waspl*) (Figure S13). Furthermore, an extensive analysis of hemoglobin levels in primitive erythroid cells at 2 dpf showed that, with the exception of *brd3a*, *kalrn2 and kif1b*, primitive erythropoiesis was largely unaffected following MO knock down of candidate genes (Figure S14). These results are consistent with the notion that the majority of candidate genes are dispensable for specification and differentiation of primitive erythrocytes and that fundamentally different molecular mechanisms regulate primitive and definitive erythropoiesis.

To establish the role of candidate genes in differentiation of the myeloid lineage, that is neutrophils and macrophages, we performed Sudan Black staining (for neutrophils) and *in situ* hybridization using *mpeg* riboprobe (for macrophages) in control and candidate gene depleted zebrafish embryos at 3 dpf. Out of 15 genes tested, depletion of nine resulted in reduced numbers of Sudan Black positive cells and two (*kif1b*, *waspla*) had an effect on the number of macrophages (Figures S15, S16, and S18). Reduction in the number of Sudan Black positive cells could reflect the absence of granules rather than neutrophils. We have, therefore, performed *in situ* hybridization using *mpx* riboprobe for all genes for which the knockdown resulted in a decrease in the number of Sudan Black positive cells. For all the tested genes the observed phenotype was comparable to the one we reported following Sudan Black staining (Figure S17).

Finally, we analyzed the impact of loss of candidate gene function on lymphoid development. Differentiated thymic T-cells are exclusively derived from definitive HSCs and can be readily identified by *rag1* expression when examined at 4 dpf (Figure S19). Not surprisingly, a significant decrease in *rag1* staining was mostly observed in the same set of genes in which we observed a decrease in *c-myb* staining (a marker for HSCs), namely *kalrn1*, *kalrn2*, *mfn2*, *pdia5* and *psmd13*. In addition, injection of *akap10* MO and *rcor1* MO, which had no negative impact on the number of *c-myb* positive cells, resulted in a significant decrease in the number of T lymphocytes.

### Analysis of expression patterns for phenotype classification

**Figure 3:**
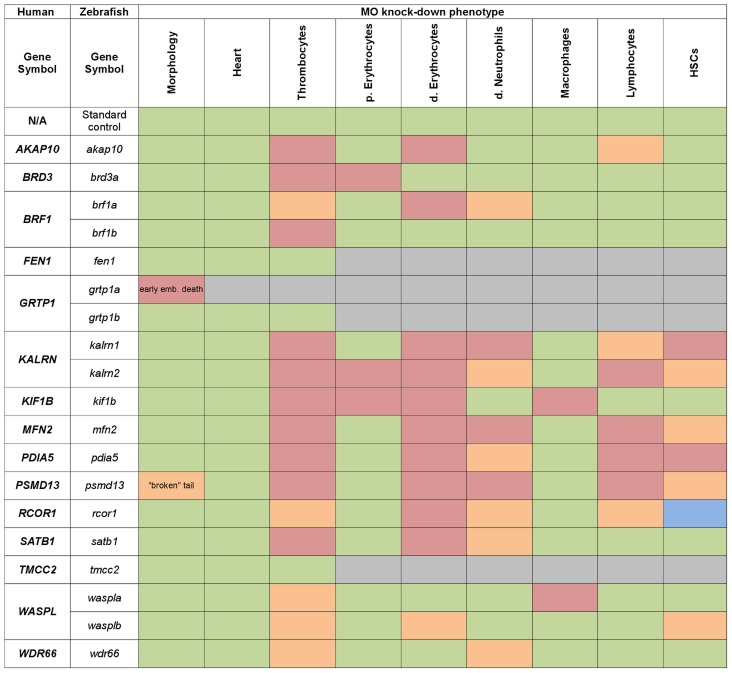
Heat map summarising hematopoietic phenotypes of knock-down of 19 candidate genes. Data obtained from the initial knock-down were used to generate a heat map of phenotype profiles. Each colored cell in the heat map shows the severity of the observed phenotype relative to control. The most severe decrease in the number of cells is displayed in red, a moderate reduction is displayed in orange and green denotes a cell number comparable to control. A moderate increase in the cell number is displayed in blue and grey indicates that the test was not performed.

**Figure 4:**
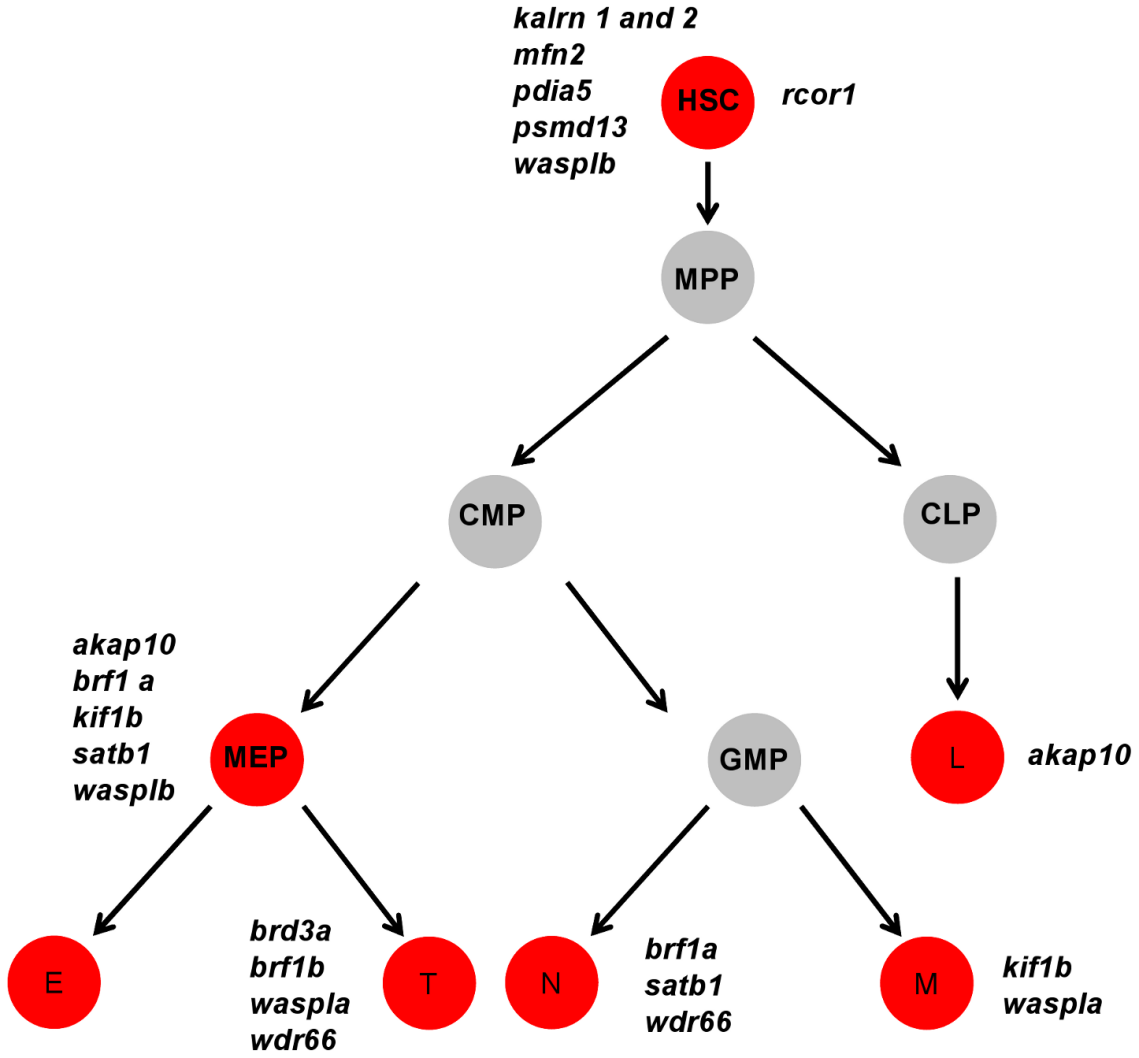
A cartoon model of the hematopoietic tree showing the stages most likely affected by candidate gene knockdown. Based on the heat map of phenotype profiles we have hierarchically positioned candidate genes on the hematopoietic tree and assigned them a potential role during hematopoietic differentiation. Analysis of the hematopoietic tree identified a set of genes enriched in the HSC cluster, namely: *kalrn 1* and *-2*, *mfn2*, *pdia5*, *psmd13*, *rcor1* and *wasplb* and another set in the thrombocyte-erythroid progenitor cell cluster, namely: *akap10*, *brf1a*, *kif1b*, *satb1*, *wasplb*. MPP – multipotent progenitor cell, CMP – common myeloid progenitor, CLP – common lymphoid progenitor, MEP – thrombocyte-erythroid progenitor, GMP – granulocyte-macrophage progenitor, L – lymphocyte, E – erythrocyte, T – thrombocyte.

The large number of genes analyzed and the resultant volume of data acquired can present challenge in understanding and interpreting the results. Hence, we used the information gained from the initial MO knockdown screen to generate a heat-map of phenotype profiles (Figure 3) and cluster genes with similar phenotypic profiles. We then hierarchically positioned candidate genes on the hematopoietic lineage tree and assigned each of them a potential role during hematopoietic differentiation (Figure 4).

Our analysis of the hematopoietic lineage tree revealed a distinct pattern of gene distribution, suggesting two main gene clusters. The first cluster represents a set of genes, namely *kalrn1* and -*2*, *mfn2*, *pdia5*, *psmd13*, *rcor1* and *wasplb*, which upon depletion affect the number of HSCs. The second major cluster represents a set of genes, namely *akap10*, *brf1a*, *kif1b*, *satb1* and *wasplb*, which appear to be essential further down the hematopoietic tree and affect differentiation of both definitive erythrocytes and thrombocytes. These genes have presumed role in HSC fate decisions prior to specification of the thrombocyte and erythrocyte progenitors.

Although the frequency of blood defects observed in our screen was high, the screen was not as well suited for the identification of knockdown phenotypes that result in subtle differences in myeloid lineage cell production or skewing of myeloid lineage differentiation. This is mainly because changes in the number of neutrophils and macrophages arising from HSCs may be undetectable using markers and the developmental time point outlined here. Previous studies have shown that erythroid-myeloid progenitor cells (EMPs) are capable of generating both macrophages and neutrophils and that these blood cells appear in *mib* zebrafish despite the absence of HSCs [9]. However, even with these caveats, we believe the screening procedure used has proven effective for extracting functional information from a GWAS dataset in a medium-throughput manner.

### *brd3a* is a rate limiting regulator of thrombopoiesis

**Figure 5:**
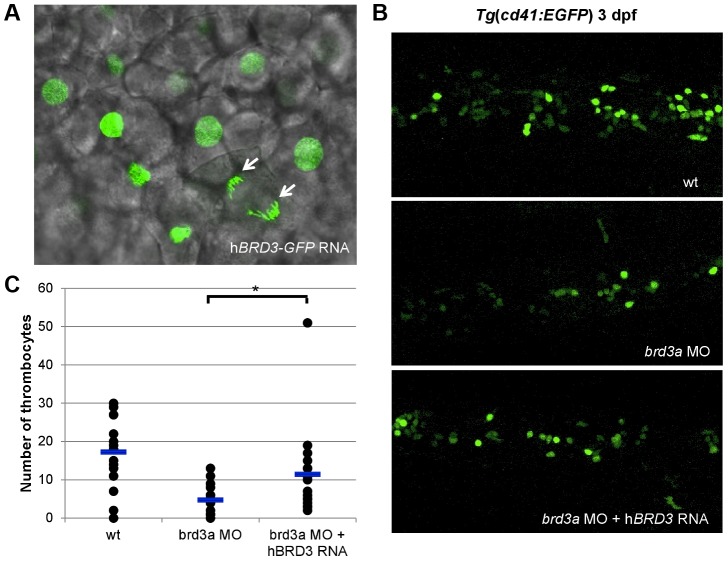
*brd3a* is an important regulator of thrombopoiesis. (A) Live confocal imaging of zebrafish embryos injected with h*BRD3-*GFP mRNA revealed the nuclear localization of h*BRD3* and that it binds to mitotic chromosomes (white arrows). Expression of h*BRD3* in *brd3a* MO injected embryos resulted in a partial rescue of the number of thrombocytes as shown in (B). (C) A graph to illustrate the number of thrombocytes in control, splice *brd3a* MO and splice *brd3a* MO plus h*BRD3* mRNA injected embryos. Each dot represents the number of thrombocytes in the individual MO-injected embryos with respect to control. A blue horizontal line represents the mean value of the number of thrombocytes for each group of embryos. Student t test, * p = 0.016; n = 17. All embryos are oriented with anterior to the left and dorsal to the top.

To gain an additional insight into mechanisms by which these newly discovered genes affect thrombopoiesis, we performed a more extensive analysis of the function of *brd3a* in hematopoiesis. The bromodomain and extra terminal domain (BET) family of proteins, including BRD2, BRD3, and BRD4, are evolutionally conserved and play a key role in many cellular processes by controlling the assembly of histone acetylation-dependent chromatin complexes [10]. To further confirm that the defects observed in the *brd3a* depleted embryos resulted from loss of *brd3a*, *in vitro*–transcribed RNA encoding human *BRD3* (h*BRD3*) was injected into 1-cell stage embryos. Live confocal imaging of zebrafish embryos injected with h*BRD3-*GFP confirmed that hBRD3 binds to mitotic chromosomes (Figure 5A), a feature previously reported for BET family proteins i.e. BRD2, BRD3 and BRD4 [11]–[13]. Expression of h*BRD3* in *brd3a* MO injected embryos resulted in partial but statistically significant rescue of the number of thrombocytes demonstrating that *brd3a* MO used in this study exerted a specific effect (Figure 5B, C).

### *brd3a* is essential for differentiation and not maintenance of thrombocytes

Morpholinos do not allow gene-specific perturbation to be carried out with temporal resolution, which is a disadvantage when dissecting the precise role of a selected gene in hematopoiesis. A number of studies reported that compounds targeting BET proteins might be used to manipulate hematopoietic development for exploratory or therapeutic purposes [14]–[16]. The BET family inhibitor, thieno-triazolo-1,4-diazepine ((+)-JQ1 in short) is a potent, highly specific inhibitor which displaces BET proteins from chromatin by competitively binding to the acetyl-lysine recognition pocket of BET bromodomains [17], [18]. Thus, we evaluated the pharmacological impact of (+)-JQ1 on zebrafish development and thrombopoiesis. Exposure of zebrafish embryos to (+)-JQ1 disrupted the chromatin occupancy of h*BRD3*-GFP confirming the efficacy of the inhibitor (Figure S20 A–C).

**Figure 6:**
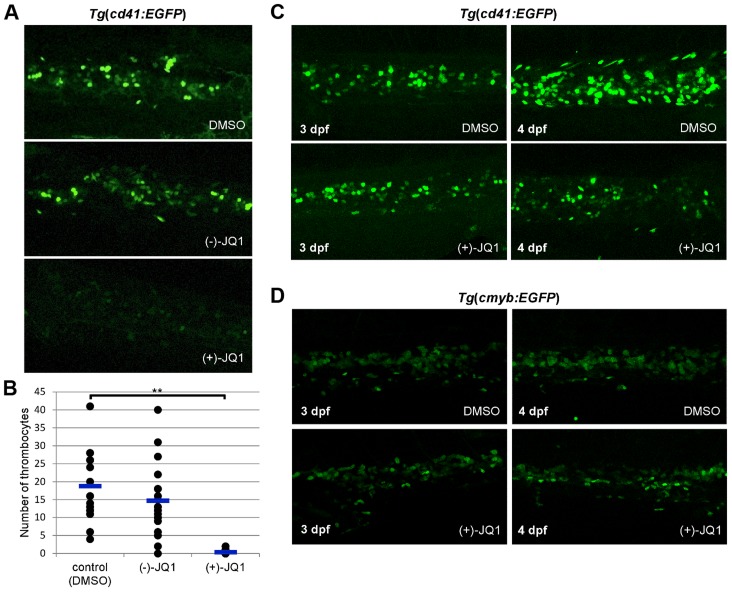
*brd3a* is required for differentiation and not maintenance of thrombocytes. (A) Exposure of zebrafish embryos to 1 µg/ml (+)-JQ1 from 24 hpf resulted in a severe reduction in the number of thrombocytes compared to DMSO or (−)-JQ1 treated embryos. (B) A graph to illustrate the average number of thrombocytes at 3 dpf. Embryos were exposed to DMSO, (−)-JQ1 or (+)-JQ1 starting from 24 hpf. Each dot represents thrombocyte count for a single embryo. Horizontal line denotes average thrombocyte number. Student t test, ** p = 1.6×10^−8^, n = 17. (C) To assess whether *brd3a* is essential for differentiation of thrombocytes or for their maintenance and survival we incubated *Tg(cd41:EGFP)* embryos in DMSO or with (+)-JQ1 inhibitor starting from 3 dpf and checked thrombocyte number 24 hours later, at 4 dpf. We found that in untreated (DMSO) embryos the number of thrombocytes markedly increased between 3- and 4 dpf; however, in (+)-JQ1 treated embryos there was no change in the number of thrombocytes. In contrast, the inhibitor had no adverse effect on the number of HSCs during this 24 h period of treatment (D). Representative confocal images of CHT region are shown. All embryos are oriented with anterior to the left and dorsal to the top.

We next incubated embryos from 6 hpf in various concentrations of (+)-JQ1 and (−)-JQ1 (stereoisomer which has no appreciable affinity to BET bromodomains) [17] as a control (Figure S21). Exposure of zebrafish embryos to 1 µg/ml (+)-JQ1 resulted in complete mortality by 24 hpf whereas the (−)-JQ1 enantiomer showed no observable effect on embryo development (Figure S21). This early embryonic death of zebrafish embryos was not surprising considering that knockout of *Brd2* or *Brd4* in mice results in embryonic lethality, indicating an important role of these two proteins in embryonic development [19], [20]. When treated, however, with (+)-JQ1 from 24 hpf, embryos exhibited overall normal development even at the higher concentration (1 µg/ml) of (+)-JQ1 (Figure S21). Although morphologically normal, thrombopoiesis was completely abolished in these embryos (Figure 6 A). Interestingly, the decrease in the number of thrombocytes appeared more prominent in the presence of (+)-JQ1 inhibitor compared to *brd3a* MO knock down. This opened the possibility that other members of the BET family might be contributing to the observed phenotype. To investigate this further, we performed MO knock down of zebrafish *brd2a*, *brd2b* and *brd4* and assessed the number of thrombocytes at 3 dpf. Single MO knock down of all three genes resulted in a severe decrease in the number of thrombocytes. These data strongly suggested that, indeed, other members of BET family of proteins (i.e. *brd2* and *brd4*) play an important role in thrombopoiesis (Figure S22 A–C).

Both MO knock down and treatment with (+)-JQ1 inhibitor from 24 hpf resulted in a severe reduction in the number of thrombocytes at 3 dpf, suggesting an essential role for *brd3a* in the differentiation of thrombocytes as opposed to a requirement for their maintenance and survival. To address this question we incubated *Tg(cd41:EGFP)* embryos with (+)-JQ1 inhibitor starting from 3 dpf, when there is already a considerable number of thrombocytes in CHT, and assessed their number 24 hours later, at 4 dpf. We found that in untreated and (−)-JQ1 treated embryos the number of thrombocytes markedly increased between 3 and 4 dpf. However, in (+)-JQ1 treated embryos we observed no change in the number of thrombocytes (Figure 6C). Moreover we found that (+)-JQ1 had no adverse effect on the number of HSCs during this 24 h period of treatment (Figure 6D). Taken together this strongly suggests that *brd3a* is important in differentiation of thrombocytes from HSCs, however, once differentiated, *brd3a* was dispensable for their maintenance and survival.

## Discussion

GWAS meta-analysis of platelet size and number has been successful in identifying SNPs associated with the mass (volume x count) of platelets. In contrast with the results of GWAS in common diseases, more than 80% of SNPs associated with hematological traits are localized within 10 Kb of genes providing a sound argument to infer biologically relevant candidate genes [3], [4]. Canonical pathway analyses detected a highly significant over-representation of “core genes” (the sentinel SNP is within the gene or within 10 kb from the gene) in relevant biological functions such as hematological disease, cancer and cell cycle [3]. However, three quarters of regions proximal to the platelet GWAS SNPs harbor unfamiliar genes or known genes not previously implicated in hematopoiesis that merit extensive follow-up analysis. Therefore, this study was set up to address the need for a medium-throughput method in zebrafish to dissect the functional roles of these assumed novel regulators of hematopoiesis.

In total, our screen identified 15 genes (corresponding to 12 human genes) required for distinct stages of specification or differentiation of HSCs in zebrafish. A detailed review of the content of databases and literature revealed limited knowledge about the functional role of *Satb1*, *Rcor1* and *Brd3* [21]–[24] in hematopoiesis and for the remaining nine genes our work represents the first study on their putative role in hematopoiesis. Importantly, our results are well in line with some of the findings reported by others. One example is *RCOR1* - lineage-restricted deployment of RCOR1 and LSD1 cofactors, through interaction with Gfi proteins, controls hematopoietic differentiation [23]. Knock down of *rcor1* in zebrafish resulted in completely blocked differentiation of erythroid, thrombocytic, myeloid and lymphoid lineages. These findings strongly support the hypothesis that the published platelet GWAS [3] enriched for functional regulators of the hematopoiesis and further support previous assumptions that a large proportion of the genes uncovered by the aforementioned GWAS also have a conserved role in zebrafish.

In this study, we followed a two step screening approach: in the first instance, we used the *Tg(cd41:EGFP)* line in conjunction with a panel of hematopoietic *in situ* hybridization probes and histochemical staining to create a heat map with distinct “phenotype signatures” of each gene knock-down. We then positioned the candidate genes on the hematopoietic cell lineage tree and assigned them a potential role in hematopoietic differentiation. Interestingly, our screen revealed that, although initially selected based on their effect on the platelet size and/or number, none of the candidates exerted a thrombocyte specific effect. These results should be interpreted within the context of several major differences between the effect of the SNPs and whole embryo MO knock down on hematological traits.

First, the majority of associated SNPs identified in platelet GWAS are in non-transcribed regions and it is likely that the underlying mechanism linking them to the phenotype is regulatory. Thus, the functional effects of SNPs are subtler compared to the knock down of transcripts achieved by MOs in our screening. Secondly, although GWAS provided a list of SNPs associated with the platelet size and number, there is no evidence about the biological processes that link the associated SNP to the phenotype. Indeed, it has been shown that in most cases the reported SNP is not the functional SNP itself but is in linkage disequilibrium with the SNP overlapping a functional region [25]. Experimental evidence shows that open chromatin profiles of megakaryocytes and erythroblasts differ and thus cell type-restricted regions of open chromatin could influence the penetrability of the functional SNP [26], [27] in a lineage specific manner. In contrast, MO knock down in zebrafish is not spatially restricted and thus offers the opportunity to determine the functional role of candidate genes in all blood lineages.

To further verify the hematopoietic role of genes identified in GWAS, we performed a more extensive evaluation of the effect of *brd3a* on thrombopoiesis. It has been shown that BRD3 interacts with acetylated GATA1 and stabilizes its chromatin occupancy [21]. A pharmacologic compound, JQ1, that occupies the acetyl-lysine binding pockets of Brd3 bromodomains disrupts the BRD3-GATA1 interaction, diminishes the chromatin occupancy of both proteins, and inhibits erythroid maturation [21]. Although GATA1 and BRD3 co-occupancy on GATA1 target genes was also observed in a megakaryocytic cell line [21], the biological relevance of this binding was never confirmed. Here we report on the important role of *brd3a* in thrombopoiesis. Indeed, knock-down of *brd3a* with two independent MOs as well as treatment of zebrafish embryos with the JQ1 inhibitor starting from 24 hpf severely reduced the number of thrombocytes in 3 days old embryos. Interestingly, incubation of embryos with JQ1 inhibitor between 3- and 4 dpf, that is after the onset of thrombopoiesis, did not have any effect on the already differentiated thrombocytes. However, the number of thrombocytes failed to increase compared to control embryos within this 24-hour period. These results strongly support the idea that *brd3a* is critical for establishing but not maintaining thrombopoietic compartment. Some previous studies suggest that BRD3, as well as some other mitotically retained factors, functions as a molecular “bookmark” by enabling post-mitotic transcription re-initiation of its target genes [13], [28]. It is plausible to assume that a similar mechanism is employed during thrombopoiesis. In that scenario, retention of Brd3 on chromatin during mitosis of thrombocyte precursors or erythroid-thrombocyte progenitor cells would contribute to the maintenance of transcription patterns necessary for establishment of thrombocyte identity. However, further work will be necessary to identify the precise molecular mechanisms by which *brd3a* exerts its effect on thrombopoiesis. Taken together, our study provides a paradigm of the usefulness of zebrafish for efficient translation of GWAS findings into relevant biological information in an objective and unbiased manner. GWAS have mapped many novel, convincingly associated loci in the proximity of genes where functional significance is expected. So far, functional validation of such genes has remained confined to single gene approaches. Here we utilized the powerful genetics and translucency of zebrafish larvae to undertake a medium-throughput screen of genes implicated in human hematopoietic variation. The results of this screen will help us to tentatively place novel genes in molecular pathways and thus close the ever-increasing knowledge gap on the biological function of gene candidates identified by genomic technologies.

## Materials and Methods

### Ethics statement

The maintenance, embryo collection and staging of the wild type (Tubingen Long Fin) and transgenic zebrafish lines (Tg(*cd41:GFP*), Tg(*fli1:GFP*), Tg(*c-myb:EGFP*) were performed in accordance with EU regulations on laboratory animals, as previously described [29], [30].

### Embryo injections

Morpholinos (GeneTools, LLC) were re-suspended in sterile water and diluted to chosen concentration. Approximately 1 nl was injected into embryos at 1- to 2-cell stage. MOs used are summarized in Table S2.

Plasmid with full-length human *hBRD3* cDNA was purchased from Source Bioscience (Nottingham, UK). *hBRD3* was cloned into pCS2 expression vector using gene-specific primers: AATTACATCGATACCATGTCCACCGCCACGACAGT (forward) and CCCGAGTCTAGACTATTCTGAGTCACTGCTGTCAGA (reverse) and AAATTAGAATTCACCATGTCCACCGCCACGACAGT (forward) and ATGTTAACCGGTAGTTCTGAGTCACTGCTGTCAGA (reverse) for cloning into the pCS2-EGFP vector. Restriction enzyme sites (ClaI/XbaI and EcoRI/AgeI respectively) used for cloning are underlined. Zebrafish full-length *rcor1* cDNA was cloned into pCS2 vector using gene-specific primers: GTTATAGAATTCATGCCCGCAATGTTAGAGAAG (forward) and AGGCGCCTCGAGTCAGGAAACCGAAGGGTTCTG (reverse). Restriction enzyme sites (EcoRI and XhoI, respectively) are underlined.

*hBRD3*, *hBRD3-GFP* and *rcor1* mRNA was synthesized with mMESSAGE mMACHINE kit (Ambion), according to the manufacturer's protocol. In the rescue experiment, 100 pg *of hBRD3* mRNA or 125 pg *rcor1* RNA was injected into the one cell stage control and MO-injected *Tg*(*cd41:EGFP*) embryos. In the hBRD3 localization experiment, 300 pg of *hBRD3-GFP* mRNA was injected into Tubingen Long Fin embryos at 1-cell stage.

### Reverse Transcription-Polymerase Chain Reaction (RT-PCR)

In order to verify the effectiveness of MOs in affecting their target transcripts, RT-PCR was performed. RNA was subjected to reverse transcription using Superscript II Reverse Transcriptase (Invitrogen). PCR was performed using gene-specific primers (listed in Table S2) and KOD Hot Start DNA Polymerase (Novagen).

### Whole mount *in situ* hybridisation

*In situ* hybridization was performed with riboprobes specific for *c-myb*, *αe1-globin*, *mpx*, *mpeg* and *rag1* as previously described [31], as well as for *brd3a*, *brf1b*, *kalrn1*, *waspla* and *wdr66*. Primers used for PCR amplification of candidate genes for probe synthesis are listed in Table S3. Photomicrographs were taken with a Zeiss camera AxioCam HRC attached to a LeicaMZ16 FA dissecting microscope (Leica Microsystems, Germany) using the AxioVision software.

### O-dianisidine staining

O-dianisidine staining was performed as previously described [32].

### Sudan Black staining

Sudan Black staining was performed as previously described [33].

### Clotting time assay

Clotting time assay was performed as previously described [7]. In short, 5 dpf larvae were anaesthetized in 0.02% tricaine solution in embryonic water and transferred onto a Petri dish in a small drop of liquid. Caudal veins of the larvae were wounded with the tip of a Microlance needle (0.4 mm×13 mm, Becton Dickinson) in the anal area. For each larva the time passing between inflicting the wound and the stop of bleeding was recorded.

### Genotyping

Genomic DNA was isolated from 5 dpf larvae, which were individually loaded into wells of a 96-well plate. Fish were incubated in 25 µl of lysis buffer (25 mM NaOH with 0.2 mM EDTA) at 95°C for 30 min. Afterwards 25 µl of neutralization buffer (40 mM Tris-HCl) was added. Genotyping was carried out using the KASP genotyping assays (KBioscience). Each reaction consisted of 4 µl of genomic DNA and 5 µl of PCR mix, according to the manufacturer's protocol (KBioscience). PCR products were analyzed using PHERAstar plus (BMGlabtech) and KlusterCaller software (KBioscience).

### Imaging

Images were captured with the use of a Leica TCS SP5 confocal microscope with Leica LAS AF software (Leica Microsystems), using a 40× immersion lens or with Axio Zoom.V16 fluorescent microscope with AxioCam MRm camera using 260× magnification.

### BET protein inhibitor

Selective inhibitor of human BET family of bromodomain-containing proteins, thieno-triazolo-1,4-diazepine, named JQ1, was kindly provided by Dr Chas Bountra, Structural Genomics Consortium, University of Oxford, Oxford, UK. Both the active inhibitor, (+)-JQ1, and its inactive stereoisomer, (−)-JQ1, were dissolved in dimethylsulfoxide (DMSO) to 10 mg/ml and stored in aliquots at −20°C. For zebrafish embryo treatment, (+)-JQ1 and (−)-JQ1 were diluted in egg water to desired concentration and added to the embryos at ∼6 hpf, 24 hpf or 3 dpf and afterwards replaced daily. Control embryos were incubated in the equal concentration of DMSO in egg water as inhibitor-treated embryos.

## Supporting Information

Figure S1 We selected genes with unknown function in hematopoietic biology and reliable zebrafish ortholog identification with over 38% identity at the protein level with its human counterpart. We next designed MO against each candidate gene and tested the efficacy of MOs by RT-PCR and sequencing, where appropriate. For all genes, the optimal dose of MO was selected that results in a specific phenotype but without gross lethality and defects in body shape and size, vasculature, heart and circulation. Finally, we performed phenotyping of MO injected embryos using a wide panel of different hematopoietic markers.
(TIFF)

Figure S2 Splice modifications caused by gene-specific MOs were assayed by RT-PCR, using gene-specific primers, and are seen as a band shift after gel electrophoresis of RT-PCR products. The binding site of MO to pre-mRNA is marked with a red horizontal line. For each gene a schematic diagram illustrates the effect of MOs on pre-mRNA splicing. Nucleotide sequences denote a frame shift caused by aberrant splicing. Where no band was obtained from the MO-injected group, RT-PCR for *β-actin* was used as a control for equal loading of cDNA. i-intron; e-exon.
(TIFF)

Figure S3 In order to determine the optimal dose of the MOs to be injected, a dose-response experiment was performed. For each gene the concentration of MO (shown in lower right corner of each image, in ng) was selected that elicited a specific phenotype without an overt non-specific effect. Knock-down of *grtp1a* resulted in early embryonic lethality even when injected with 0.8 ng of MO. Thus, *grtp1a* was excluded from further analysis. The optimal MO dose for each gene as determined in this experiment was used in all subsequent experiments.
(TIFF)

Figure S4 Whole-mount *in situ* hybridization using *brd3a*, *brf1b*, *waspla*, *kalrn1 and wdr66* riboprobes is shown. Panels show a lateral view of embryos at 3 dpf. All embryos are oriented with anterior to the left and dorsal to the top.
(TIFF)

Figure S5 The phenotyping was initiated by using the *Tg(cd41:EGFP)* reporter line that labels thrombocytes to identify genes in zebrafish that, when knocked down, affect thrombocyte number. The genes for which MO knock-down did not result in a change in number of thrombocytes were excluded from further analysis. For the “phenotypic” genes, we carried out a second level of analysis by performing whole mount *in situ* hybridisation for several hematopoietic markers, namely: *c-myb*, *ae1 globin*, *mpeg* and *rag1*, in order to assess the number of HSCs, definitive erythrocytes, macrophages and lymphocytes, respectively. These were complemented with the use of two histochemical stains: o-Dianisidine, to assess the number of primitive erythrocytes at 2 dpf and Sudan Black, to determine the number of neutrophils in CHT at 3 dpf. Finally, if the MO knock-down resulted in a decreased number of HSCs at 3 dpf, an additional step was introduced to assess their number at 30 hpf in AGM. Where the MO knock-down resulted in a decreased number of Sudan Black positive cells, whole mount *in situ* hybridisation for *mpx* was performed.
(TIFF)

Figure S6 The number of thrombocytes (*cd41^bright^*) in the CHT was counted for each gene knock-down. Of the 18 genes examined, knock down of 15 resulted in a 30–95% reduction in the number of thrombocytes. One-tailed Student t test was performed. Significant decrease in the number of thrombocytes was observed in: *akap10* (p = 4.47×10^−5^, n = 10), *brd3a* (p = 0.00021, n = 11), *brf1a* (p = 0.005, n = 9), *brf1b* (p = 2.5×10^−7^, n = 15), *kalrn1* (p = 1.4×10^−6^, n = 13), *kalrn2* (p = 1.2×10^−9^, n = 15), *kif1b* (p = 5.7×10^−6^, n = 17), *mfn2* (p = 1.49×10^−5^, n = 15), *pdia5* (p = 0.00018, n = 11), *psmd13* (p = 4.12×10^−8^, n = 9), *rcor1* (p = 0.00042, n = 20), *satb1* (p = 9.51×10^−7^, n = 15), *waspla* (p = 0.00157, n = 15), *wasplb* (p = 0.023, n = 20) and *wdr66* (p = 0.00027, n = 15) depleted embryos. Each dot represents the number of thrombocytes in an individual MO-injected embryo with respect to control. A blue horizontal line represents the mean value of the number of thrombocytes for each group of embryos.
(TIFF)

Figure S7 To verify that MOs used in this study exerted a specific effect on hematopoiesis we designed a second non-overlapping morpholino (MO2) for all “phenotypic” candidate genes. Splice modifications caused by gene-specific MOs were assayed by RT-PCR, using gene-specific primers, and were confirmed for all but *brf1a*, *kif1b* and *wasplb* MO2. These three MO2 were therefore excluded from further analysis. Injection of the remaining 12 MO2 resulted in the phenotype that was comparable to the one observed with the first MO. Representative fluorescent images of the CHT are shown. All embryos are oriented with anterior to the left and dorsal to the top.
(TIFF)

Figure S8 Splice modifications caused by gene-specific second non-overlapping MOs (MO2) were assayed by RT-PCR, using gene-specific primers, and are seen as a band shift after gel electrophoresis of RT-PCR products. Where no band was obtained from the MO-injected group, RT-PCR for *β-actin* was used as a control for equal loading of cDNA.
(TIFF)

Figure S9 MO activities in zebrafish embryos include both sequence-specific RNA binding as well as effects not associated with loss of function of the targeted locus i.e. “off-target” effects. As *p53* MO can reduce common off-target effects, the number of thrombocytes was assessed in CHT at 3 dpf in embryos co-injected with *p53* MO and gene-specific MOs. Concurrent knock down of *p53* with gene-specific MOs did not attenuate the thrombocyte phenotype induced by gene-specific MOs, confirming that the observed decrease in the number of thrombocytes was not induced by off-target effects of MOs. All embryos are oriented with anterior to the left and dorsal to the top.
(TIFF)

Figure S10 A) Fish heterozygous for a mutation in *brf1b* (allele sa3097, ZMP) were crossed and their progeny (n = 41) was subjected to a clotting time assay at 5 dpf. The time of clotting after caudal vein puncture was recorded and the larvae were genotyped. Each bar represents average clotting time in the group, SEM is shown. One-tailed Student t test, * p = 0.042. B) A graph to illustrate the number of thrombocytes in control (n = 33), splice *rcor1* MO (n = 28) and splice *rcor1* MO plus *rcor1* mRNA (n = 15) injected embryos. Each bar represents average number of thrombocytes in the group, SEM is shown. One-tailed Student t test, * p = 0.024. C) Representative fluorescent images of embryos in the *rcor1* rescue experiment. All embryos are oriented with anterior to the left and dorsal to the top.
(TIFF)

Figure S11 To assess the HSC emergence in the aorta-gonad-mesonephros (AGM) region, we injected gene-specific MOs in single-cell stage *Tg(c-myb:GFP)* transgenic embryos. For *kalrn1*, *mfn2*, *pdia5*, *psmd13* and *wasplb* MO injected embryos no difference in the number of HSCs was observed when compared to the control at 30 hpf. However, *kalrn2* and *rcor1* depleted embryos had a marked decrease in the number of HSCs at 30 hpf, implying an important role of these genes in specification of HSCs in the AGM. Representative images of the AGM region are shown. All embryos are oriented with anterior to the left and dorsal to the top.
(TIFF)

Figure S12 In order to assess vascular development, MOs targeting candidate genes were injected into *Tg(fli1:EGFP)* embryos. Vascular morphology of the embryos was assessed at 3 dpf. No major abnormalities in vascular morphogenesis were observed for any of the tested MOs, indicating that the hematopoietic defects were not secondary to a vascular phenotype. Representative images of the CHT region are shown. All embryos are oriented with anterior to the left and dorsal to the top.
(TIFF)

Figure S13 Whole mount *in situ* hybridization was performed using a probe specific to *αe1-globin* at 4 dpf. As a result of candidate gene knock down, depletion of *αe1-globin* staining was observed for all genes but *brd3a*, *brf1b*, *waspla* and *wdr66*. Representative images of CHT are shown. All embryos are oriented with anterior to the left and dorsal to the top.
(TIFF)

Figure S14 All the erythrocytes present in circulation of 2 dpf zebrafish embryos derive from the primitive wave of hematopoiesis. In order to assess the primitive erythropoiesis, MO injected embryos were stained with O-dianisidine at 2 dpf (arrow). Knockdown of 12 out of 15 candidate genes, namely: *akap10*, *brf1a*, *brf1b*, *kalrn1*, *mfn2*, *pdia5*, *psmd13*, *rcor1*, *satb1*, *waspla*, *wasplb* and *wdr66*, resulted in no observable phenotype. Depletion of *brd3a*, *kalrn2* and *kif1b* resulted in a severe reduction in the number of primitive erythrocytes. All the embryos are positioned anterior up and dorsal to the back.
(TIFF)

Figure S15 Individual neutrophils may be detected with Sudan Black staining. The number of neutrophils in MO-injected embryos (n_embryos_ = 15) in CHT was counted at 3 dpf. Knock-down of *akap10*, *brd3a*, *brf1b*, *kif1b*, *waspla* or *wasplb* caused no phenotype. A mild decrease (20–50%) in the number of neutrophils was observed for *brf1a*, *kalrn2*, *pdia5*, *rcor1*, *satb1* and *wdr66* MO injected embryos compared to control and *kalrn1*, *mfn2* and *psmd13* MO injected embryos showed a severe (over 50%) depletion of neutrophils when compared to control. Representative images of the CHT region at 3 dpf are shown. All embryos are oriented with anterior to the left and dorsal to the top.
(TIFF)

Figure S16 To identify neutrophils, Sudan Black staining was performed at 3 dpf. The number of neutrophils in CHT was counted in each group (n_embryos_ = 15). One-tailed Student t test was performed. Significant decrease in the number of neutrophils was observed in *brf1a* (p = 0.019), *kalrn1* (p = 3.1×10^−9^), *kalrn2* (p = 0.004), *mfn2* (p = 1.4×10^−6^), *pdia5* (p = 0.004), *psmd13* (p = 4.2×10^−11^), *rcor1* (p = 0.012), *satb1* (p = 0.011) and *wdr66* (p = 9.2×10^−4^) MO-injected embryos. Each dot represents the number of neutrophils in an individual MO-injected embryo with respect to control. A blue horizontal line represents the mean value of the number of neutrophils for each group of embryos.
(TIFF)

Figure S17 The expression of *mpx* in control as well as in *brf1a*, *kalrn1*, *kalrn2*, *mfn2*, *pdia5*, *psmd13*, *rcor1*, *satb1* and *wdr66* depleted embryos was assessed by *in situ* hybridization. For all genes tested the reduced number of *mpx* positive cells was observed when compared to the control. All embryos are oriented with anterior to the left and dorsal to the top.
(TIFF)

Figure S18 In order to detect macrophages, whole mount *in situ* hybridization was performed using the probe specific to *mpeg1* at 3 dpf. For 13 out of 15 tested MOs (*akap10*, *brd3a*, *brf1a*, *brf1b*, *kalrn1*, *kalrn2*, *mfn2*, *pdia5*, *psmd13*, *rcor1*, *satb1*, *wasplb* and *wdr66*) there was no observable difference in the number of macrophages. However, knock down of *kif1b* and *waspla* resulted in a severe reduction in the number of macrophages in CHT at 3 dpf. All embryos are oriented with anterior to the left and dorsal to the top.
(TIFF)

Figure S19 Differentiated thymic T-cells could be readily identified by *rag1* expression when examined at 4 dpf. Whole mount *in situ* hybridization with a *rag1* riboprobe revealed a severe decrease in the number of T lymphocytes in *kalrn2*, *mfn2*, *pdia5* and *psmd13* MO injected embryos in thymi (arrow) at 4 dpf. Knock down of *akap10*, *kalrn1* and *rcor1* caused a moderate decrease in T lymphocyte numbers. All the embryos are positioned anterior up and dorsal to the back.
(TIFF)

Figure S20 (+)-JQ1 is a highly specific inhibitor, which displaces BET proteins from chromatin by competitively binding to the acetyl-lysine recognition pocket of BET bromodomains. (A) Treatment of zebrafish embryos with (+)-JQ1 disrupted the chromatin occupancy of hBRD3-GFP as demonstrated by the absence of GFP-positive mitotic chromosomes in these embryos (0/142 GFP-positive cells) compared to 9/141 GFP-positive cells in the DMSO control group. (B–C) To further quantify the chromatin occupancy of hBRD3-GFP during mitosis, we selected ≥15 mitotic cells, as shown by DAPI staining, and counted how many of these cells were GFP positive in the presence of (+)-JQ1 or DMSO (control). Whereas in (+)-JQ1 treated embryos none of the DAPI positive mitotic chromosomes were GFP positive, in DMSO treated embryos 11 cells were double DAPI/GFP positive. Asterisk depicts GFP in the cytoplasm and arrow shows mitotic chromosomes.
(TIFF)

Figure S21 To assess the effect of the inhibitor on embryo development, dose response experiments were performed. Embryos treated with 1 µg/mL (+)-JQ1 from 6 hpf died by 24 hpf. The same concentration of the inactive enantiomer (−)-JQ1 did not affect the development of embryos. Incubation in lower concentration of (+)-JQ1 (0.5 µg/ml) led to aberrant development, i.e. tail malformation and heart edema. The lowest tested dose, 0.25 µg/ml, did not affect the development of the embryos. To avoid early embryonic lethality (+)-JQ1 inhibitor was added at 24 hpf. The embryos exhibited overall normal development even at the higher concentration (1 µg/ml) of (+)-JQ1. Representative images of embryo morphology are shown, taken at 24 hpf, 48 hpf and 72 hpf. All the embryos are positioned with anterior to the left and dorsal to the top.
(TIFF)

Figure S22 To assess the role of other BET family members in thrombopoiesis MO knock down of *brd2a*, *brd2b* and *brd4* was performed. A) Although morphologically normal, embryos injected with *brd2a*, *brd2b* or *brd4* MOs had a severe decrease in the number of thrombocytes at 3 dpf (B). Representative pictures of CHT are shown. C) For splice-blocking MOs the effect of the MOs was confirmed by RT-PCR. All the embryos are positioned with anterior to the left and dorsal to the top.
(TIFF)

Table S1 Selection of the genes for *in vivo* functional screening.
(TIFF)

Table S2 Sequences of morpholinos and primers used in the study. Morpholinos with no effect on the splicing of their target transcript are underlined.
(TIFF)

Table S3 Primers used for PCR amplification of candidate genes for probe synthesis.
(TIFF)
