# Supplementary figures and images for: A Loss of Function Screen of Identified Genome-Wide Association Study Loci Reveals New Genes Controlling Hematopoiesis
Source: PLoS Genet. 2014 Jul 10;10(7):e1004450. doi: 10.1371/journal.pgen.1004450 (PMC4091788; doi:10.1371/journal.pgen.1004450)

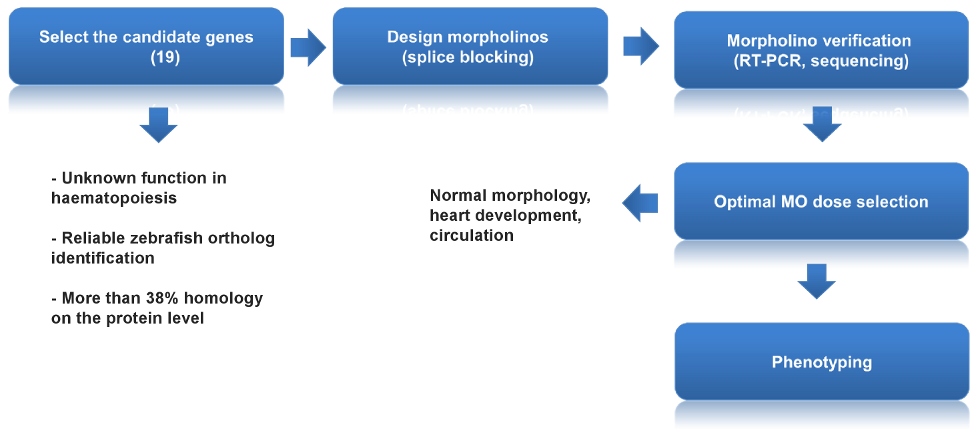

Supplement: Figure S1 — We selected genes with unknown function in hematopoietic biology and reliable zebrafish ortholog identification with over 38% identity at the protein level with its human counterpart. We next designed MO against each candidate gene and tested the efficacy of MOs by RT-PCR and sequencing, where appropriate. For all genes, the optimal dose of MO was selected that results in a specific phenotype but without gross lethality and defects in body shape and size, vasculature, heart and circulation. Finally, we performed phenotyping of MO injected embryos using a wide panel of different hematopoietic markers. (TIFF) [file pgen.1004450.s001.tiff]

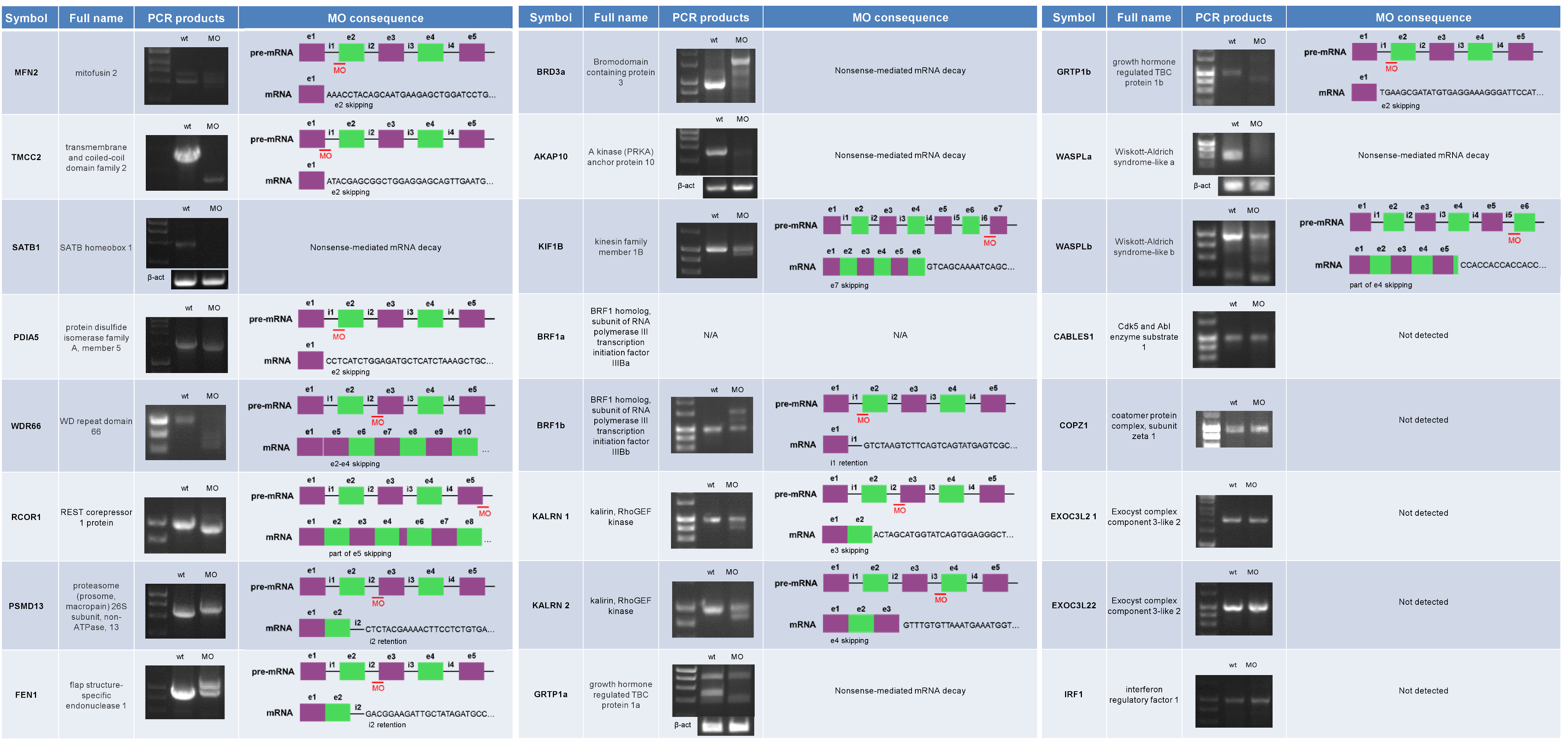

Supplement: Figure S2 — Splice modifications caused by gene-specific MOs were assayed by RT-PCR, using gene-specific primers, and are seen as a band shift after gel electrophoresis of RT-PCR products. The binding site of MO to pre-mRNA is marked with a red horizontal line. For each gene a schematic diagram illustrates the effect of MOs on pre-mRNA splicing. Nucleotide sequences denote a frame shift caused by aberrant splicing. Where no band was obtained from the MO-injected group, RT-PCR for β-actin was used as a control for equal loading of cDNA. i-intron; e-exon. (TIFF) [file pgen.1004450.s002.tiff]

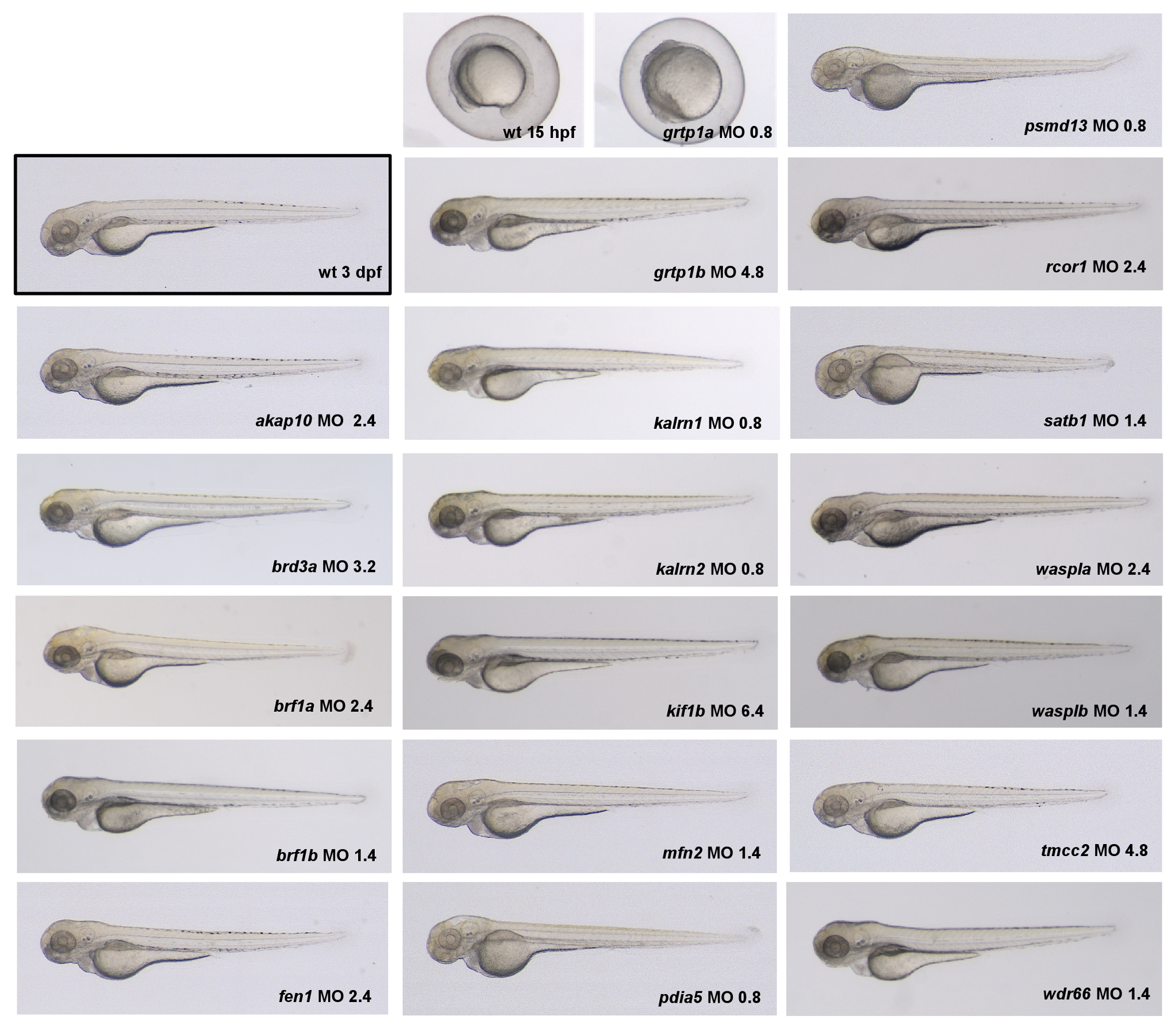

Supplement: Figure S3 — In order to determine the optimal dose of the MOs to be injected, a dose-response experiment was performed. For each gene the concentration of MO (shown in lower right corner of each image, in ng) was selected that elicited a specific phenotype without an overt non-specific effect. Knock-down of grtp1a resulted in early embryonic lethality even when injected with 0.8 ng of MO. Thus, grtp1a was excluded from further analysis. The optimal MO dose for each gene as determined in this experiment was used in all subsequent experiments. (TIFF) [file pgen.1004450.s003.tiff]

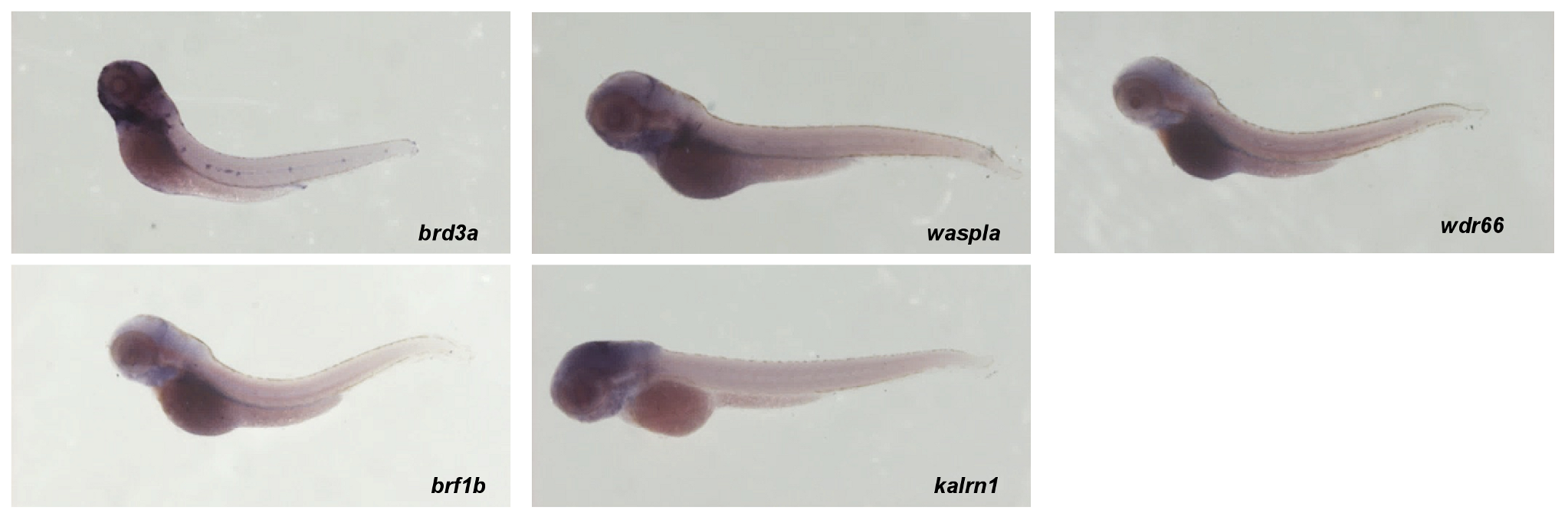

Supplement: Figure S4 — Whole-mount in situ hybridization using brd3a, brf1b, waspla, kalrn1 and wdr66 riboprobes is shown. Panels show a lateral view of embryos at 3 dpf. All embryos are oriented with anterior to the left and dorsal to the top. (TIFF) [file pgen.1004450.s004.tiff]

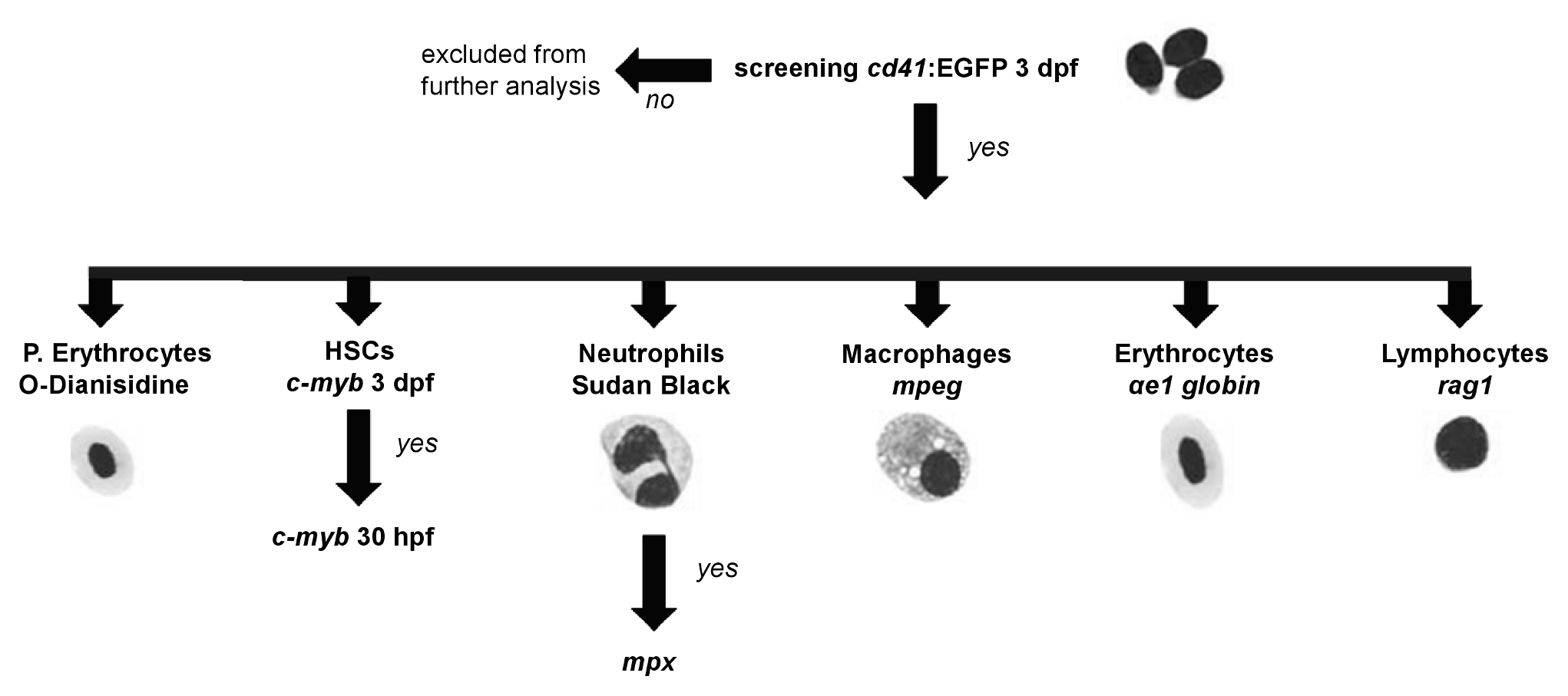

Supplement: Figure S5 — The phenotyping was initiated by using the Tg(cd41:EGFP) reporter line that labels thrombocytes to identify genes in zebrafish that, when knocked down, affect thrombocyte number. The genes for which MO knock-down did not result in a change in number of thrombocytes were excluded from further analysis. For the “phenotypic” genes, we carried out a second level of analysis by performing whole mount in situ hybridisation for several hematopoietic markers, namely: c-myb, ae1 globin, mpeg and rag1, in order to assess the number of HSCs, definitive erythrocytes, macrophages and lymphocytes, respectively. These were complemented with the use of two histochemical stains: o-Dianisidine, to assess the number of primitive erythrocytes at 2 dpf and Sudan Black, to determine the number of neutrophils in CHT at 3 dpf. Finally, if the MO knock-down resulted in a decreased number of HSCs at 3 dpf, an additional step was introduced to assess their number at 30 hpf in AGM. Where the MO knock-down resulted in a decreased number of Sudan Black positive cells, whole mount in situ hybridisation for mpx was performed. (TIFF) [file pgen.1004450.s005.tiff]

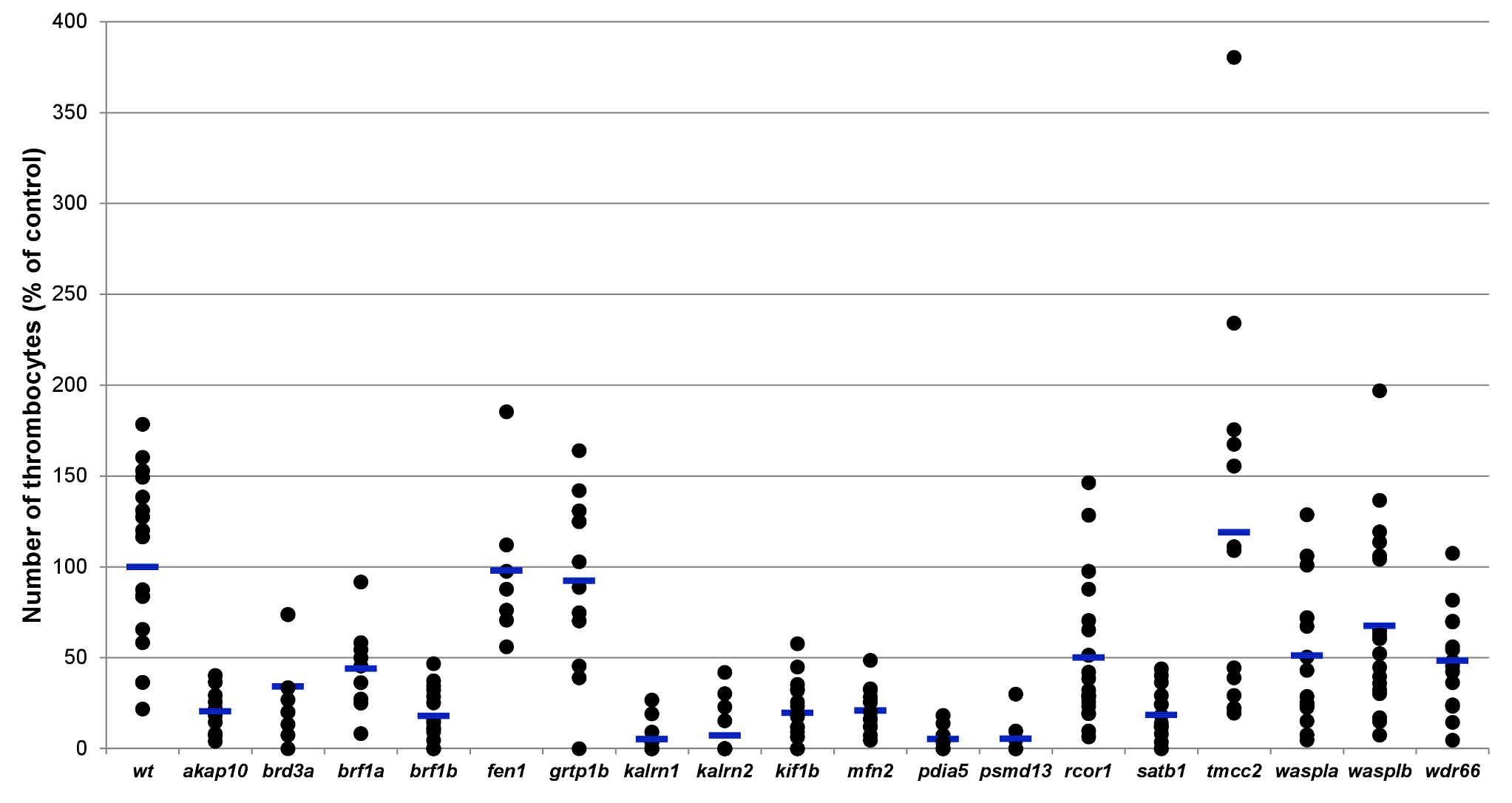

Supplement: Figure S6 — The number of thrombocytes (cd41bright) in the CHT was counted for each gene knock-down. Of the 18 genes examined, knock down of 15 resulted in a 30–95% reduction in the number of thrombocytes. One-tailed Student t test was performed. Significant decrease in the number of thrombocytes was observed in: akap10 (p = 4.47×10^−5^, n = 10), brd3a (p = 0.00021, n = 11), brf1a (p = 0.005, n = 9), brf1b (p = 2.5×10^−7^, n = 15), kalrn1 (p = 1.4×10^−6^, n = 13), kalrn2 (p = 1.2×10^−9^, n = 15), kif1b (p = 5.7×10^−6^, n = 17), mfn2 (p = 1.49×10^−5^, n = 15), pdia5 (p = 0.00018, n = 11), psmd13 (p = 4.12×10^−8^, n = 9), rcor1 (p = 0.00042, n = 20), satb1 (p = 9.51×10^−7^, n = 15), waspla (p = 0.00157, n = 15), wasplb (p = 0.023, n = 20) and wdr66 (p = 0.00027, n = 15) depleted embryos. Each dot represents the number of thrombocytes in an individual MO-injected embryo with respect to control. A blue horizontal line represents the mean value of the number of thrombocytes for each group of embryos. (TIFF) [file pgen.1004450.s006.tiff]

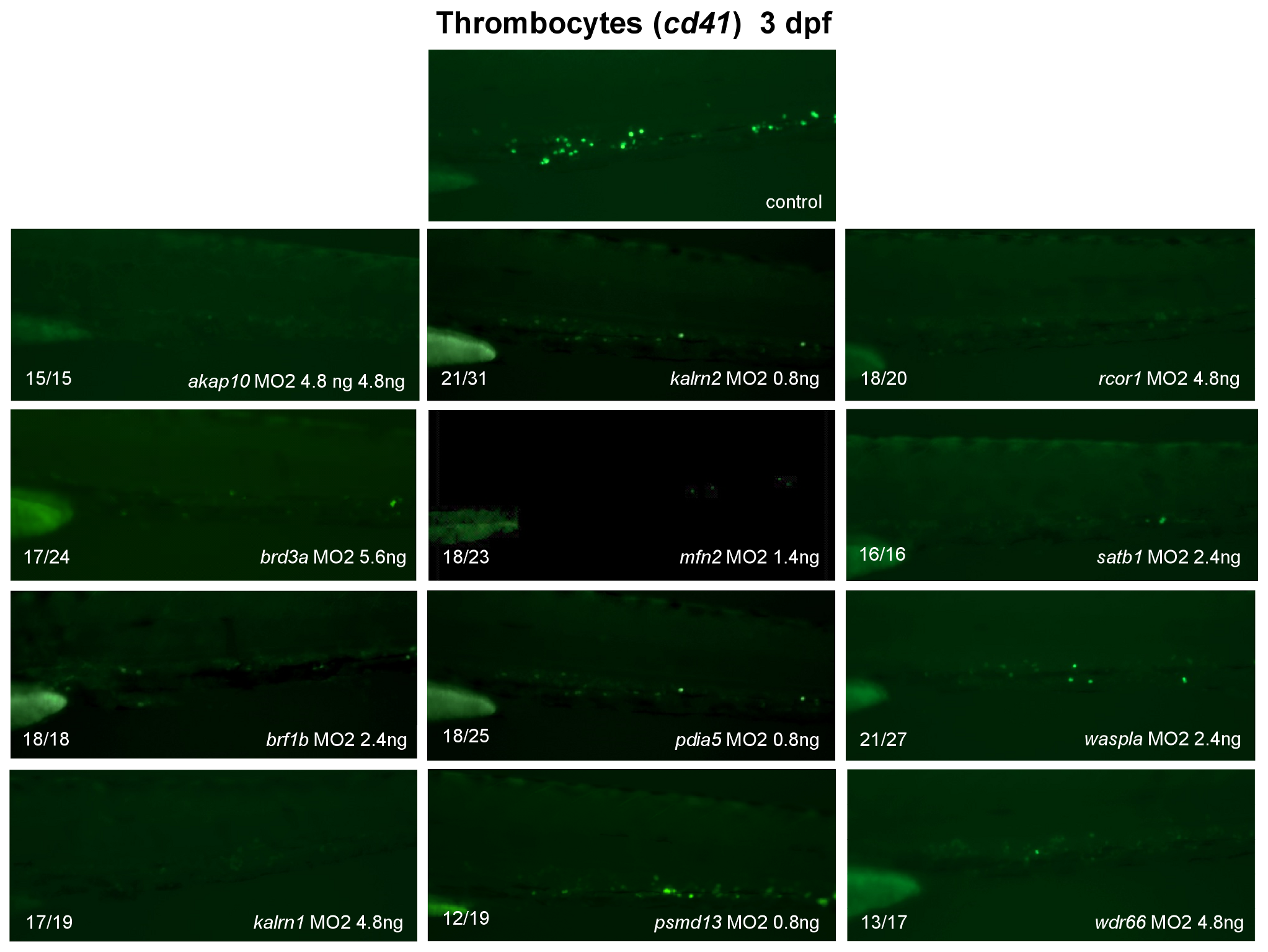

Supplement: Figure S7 — To verify that MOs used in this study exerted a specific effect on hematopoiesis we designed a second non-overlapping morpholino (MO2) for all “phenotypic” candidate genes. Splice modifications caused by gene-specific MOs were assayed by RT-PCR, using gene-specific primers, and were confirmed for all but brf1a, kif1b and wasplb MO2. These three MO2 were therefore excluded from further analysis. Injection of the remaining 12 MO2 resulted in the phenotype that was comparable to the one observed with the first MO. Representative fluorescent images of the CHT are shown. All embryos are oriented with anterior to the left and dorsal to the top. (TIFF) [file pgen.1004450.s007.tiff]

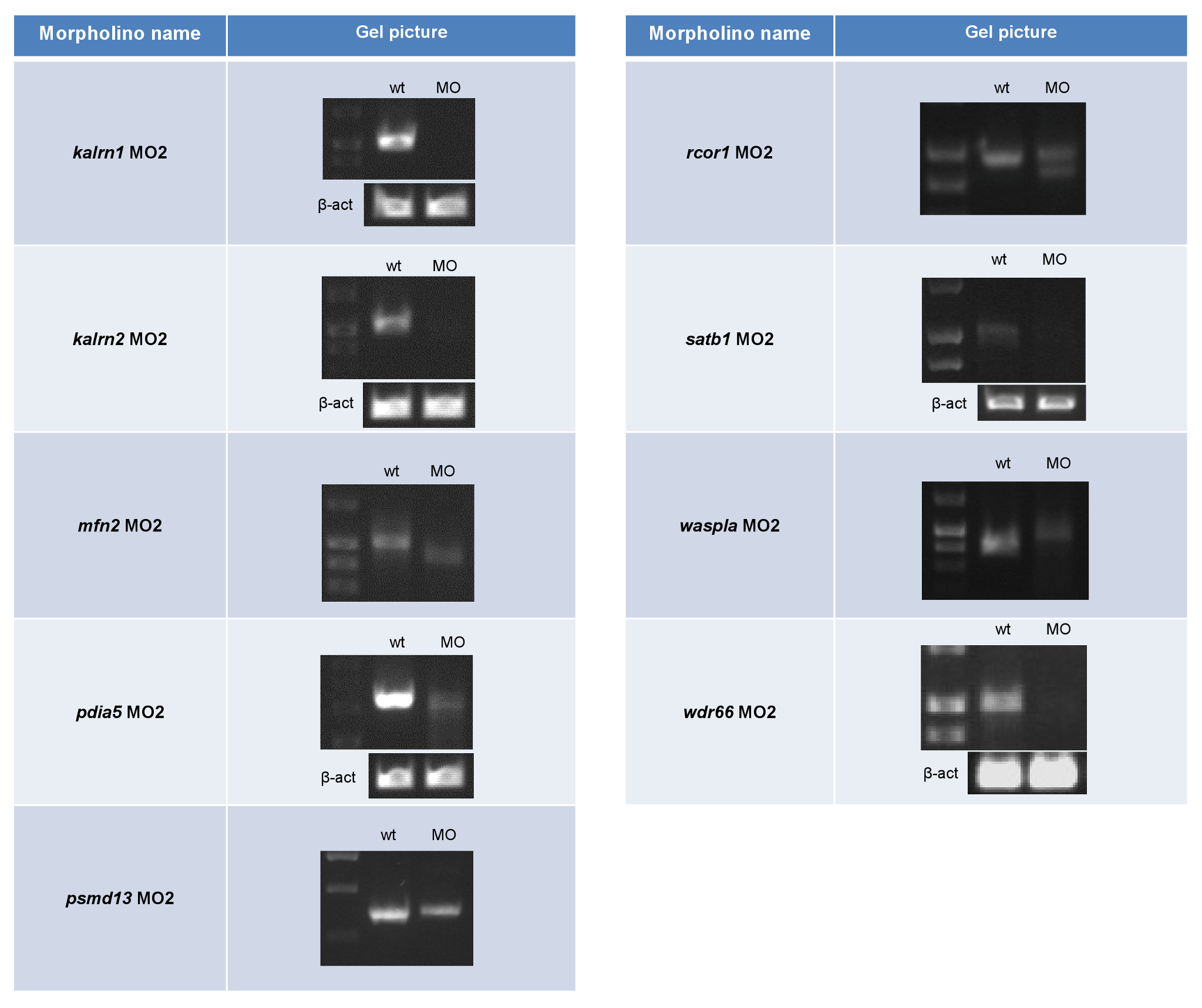

Supplement: Figure S8 — Splice modifications caused by gene-specific second non-overlapping MOs (MO2) were assayed by RT-PCR, using gene-specific primers, and are seen as a band shift after gel electrophoresis of RT-PCR products. Where no band was obtained from the MO-injected group, RT-PCR for β-actin was used as a control for equal loading of cDNA. (TIFF) [file pgen.1004450.s008.tiff]

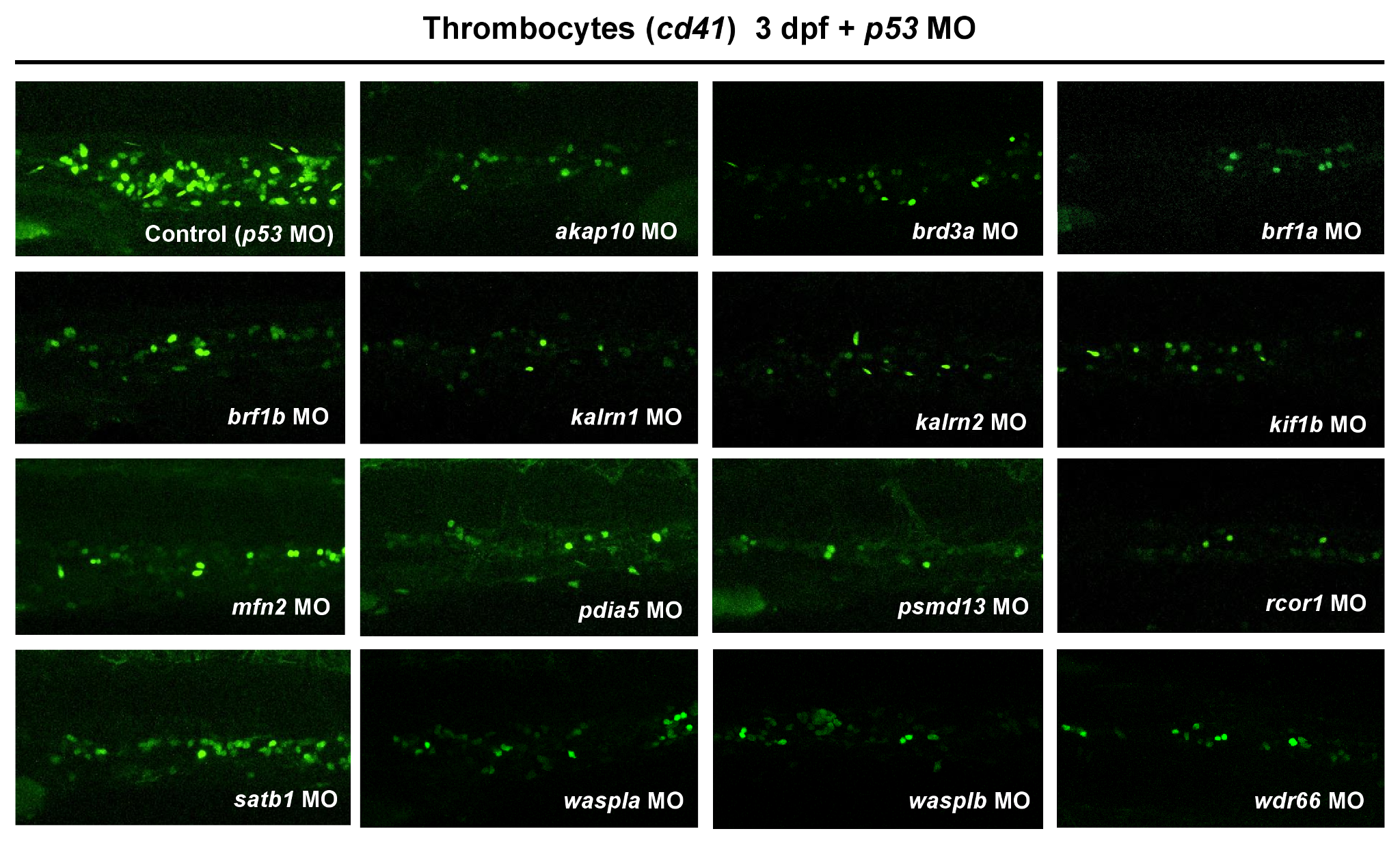

Supplement: Figure S9 — MO activities in zebrafish embryos include both sequence-specific RNA binding as well as effects not associated with loss of function of the targeted locus i.e. “off-target” effects. As p53 MO can reduce common off-target effects, the number of thrombocytes was assessed in CHT at 3 dpf in embryos co-injected with p53 MO and gene-specific MOs. Concurrent knock down of p53 with gene-specific MOs did not attenuate the thrombocyte phenotype induced by gene-specific MOs, confirming that the observed decrease in the number of thrombocytes was not induced by off-target effects of MOs. All embryos are oriented with anterior to the left and dorsal to the top. (TIFF) [file pgen.1004450.s009.tiff]

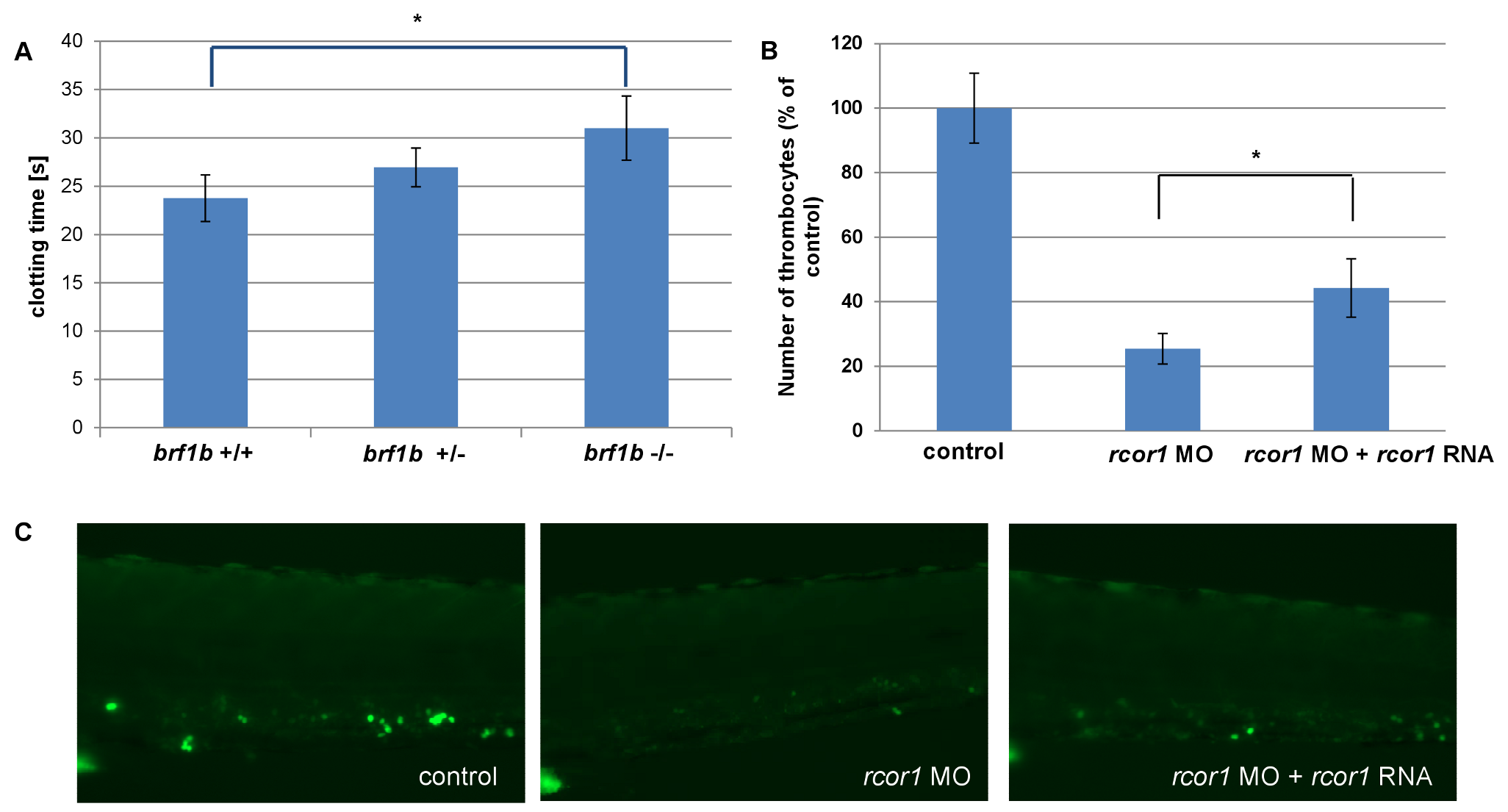

Supplement: Figure S10 — A) Fish heterozygous for a mutation in brf1b (allele sa3097, ZMP) were crossed and their progeny (n = 41) was subjected to a clotting time assay at 5 dpf. The time of clotting after caudal vein puncture was recorded and the larvae were genotyped. Each bar represents average clotting time in the group, SEM is shown. One-tailed Student t test, * p = 0.042. B) A graph to illustrate the number of thrombocytes in control (n = 33), splice rcor1 MO (n = 28) and splice rcor1 MO plus rcor1 mRNA (n = 15) injected embryos. Each bar represents average number of thrombocytes in the group, SEM is shown. One-tailed Student t test, * p = 0.024. C) Representative fluorescent images of embryos in the rcor1 rescue experiment. All embryos are oriented with anterior to the left and dorsal to the top. (TIFF) [file pgen.1004450.s010.tiff]

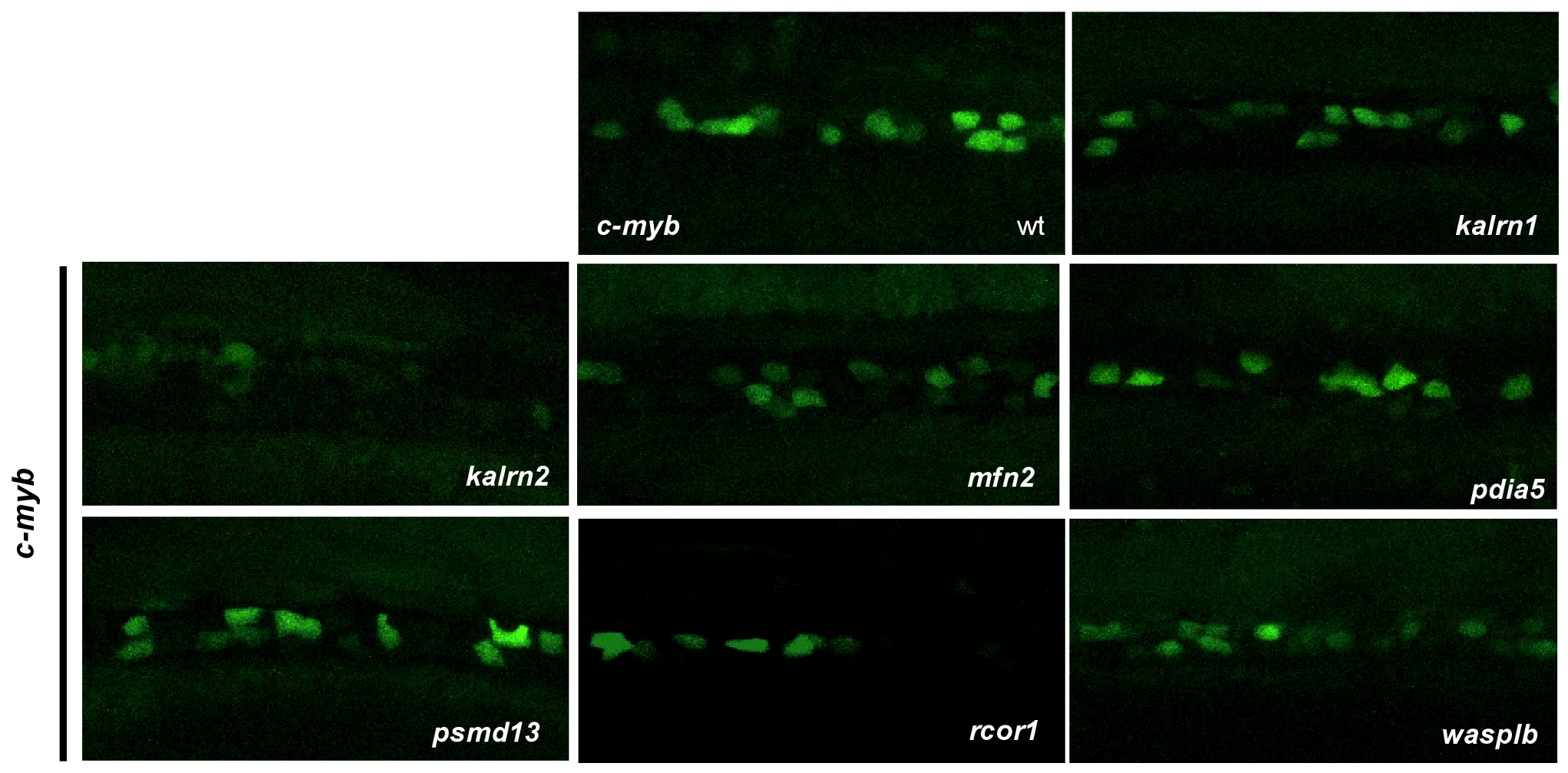

Supplement: Figure S11 — To assess the HSC emergence in the aorta-gonad-mesonephros (AGM) region, we injected gene-specific MOs in single-cell stage Tg(c-myb:GFP) transgenic embryos. For kalrn1, mfn2, pdia5, psmd13 and wasplb MO injected embryos no difference in the number of HSCs was observed when compared to the control at 30 hpf. However, kalrn2 and rcor1 depleted embryos had a marked decrease in the number of HSCs at 30 hpf, implying an important role of these genes in specification of HSCs in the AGM. Representative images of the AGM region are shown. All embryos are oriented with anterior to the left and dorsal to the top. (TIFF) [file pgen.1004450.s011.tiff]

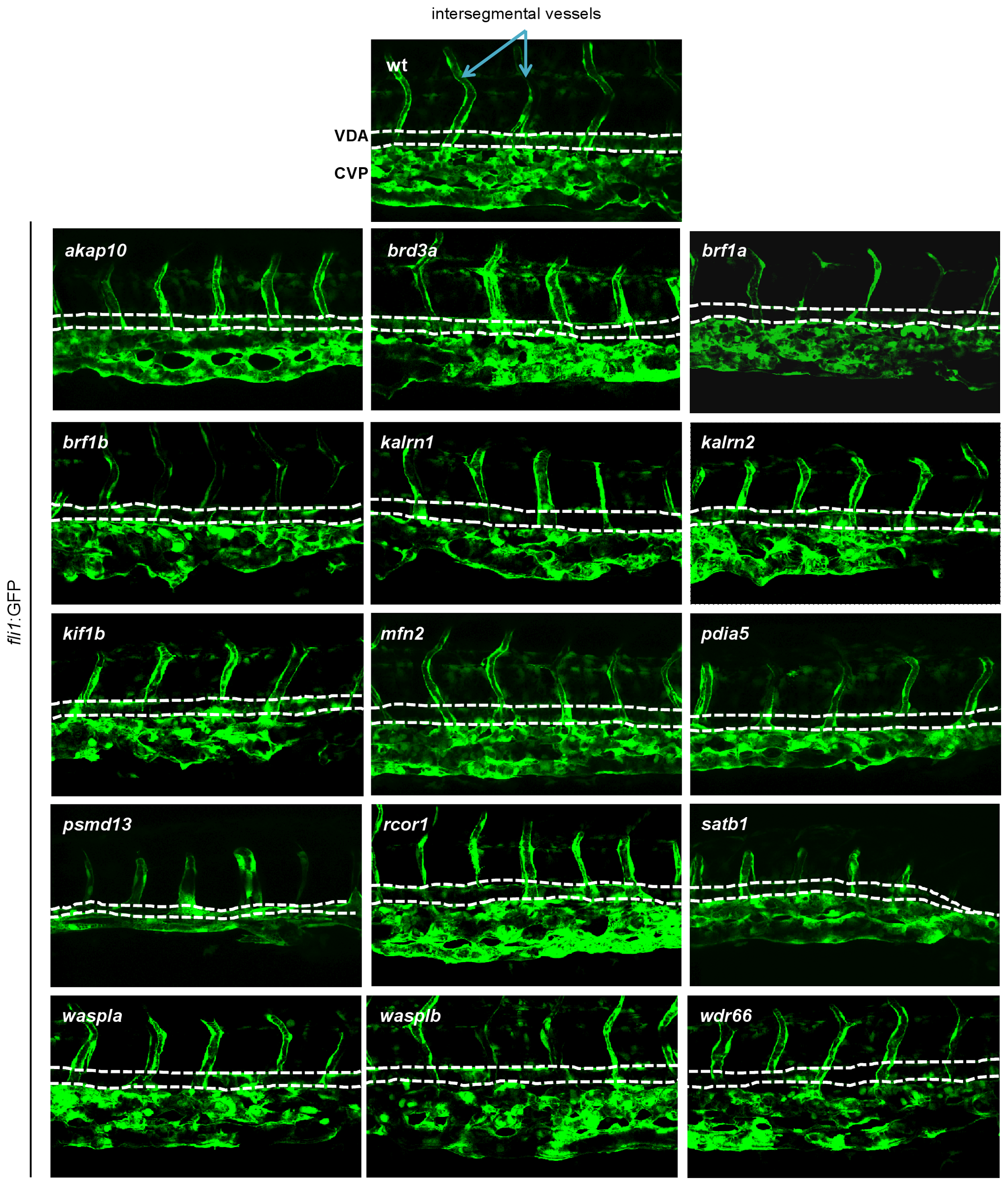

Supplement: Figure S12 — In order to assess vascular development, MOs targeting candidate genes were injected into Tg(fli1:EGFP) embryos. Vascular morphology of the embryos was assessed at 3 dpf. No major abnormalities in vascular morphogenesis were observed for any of the tested MOs, indicating that the hematopoietic defects were not secondary to a vascular phenotype. Representative images of the CHT region are shown. All embryos are oriented with anterior to the left and dorsal to the top. (TIFF) [file pgen.1004450.s012.tiff]

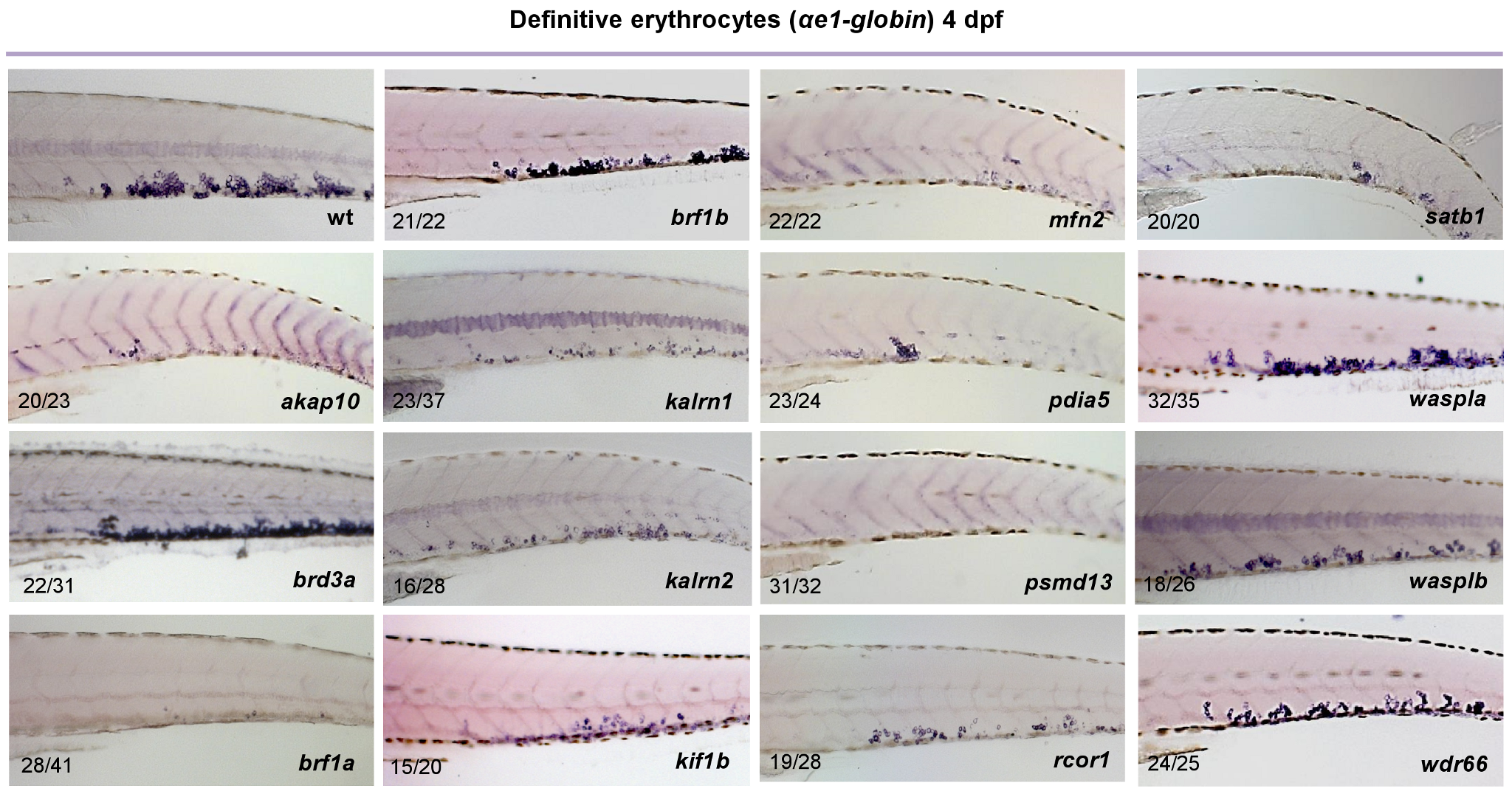

Supplement: Figure S13 — Whole mount in situ hybridization was performed using a probe specific to αe1-globin at 4 dpf. As a result of candidate gene knock down, depletion of αe1-globin staining was observed for all genes but brd3a, brf1b, waspla and wdr66. Representative images of CHT are shown. All embryos are oriented with anterior to the left and dorsal to the top. (TIFF) [file pgen.1004450.s013.tiff]

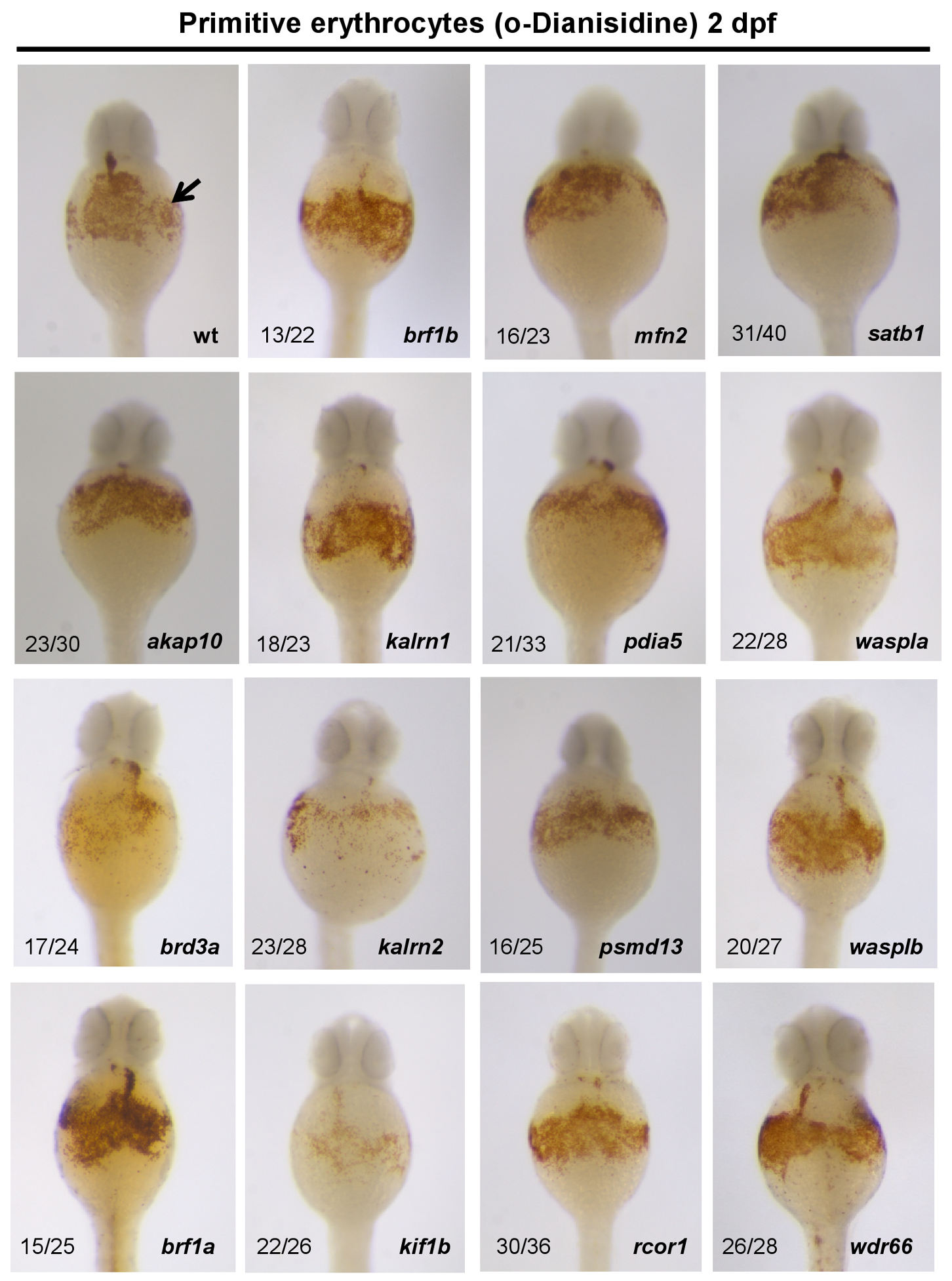

Supplement: Figure S14 — All the erythrocytes present in circulation of 2 dpf zebrafish embryos derive from the primitive wave of hematopoiesis. In order to assess the primitive erythropoiesis, MO injected embryos were stained with O-dianisidine at 2 dpf (arrow). Knockdown of 12 out of 15 candidate genes, namely: akap10, brf1a, brf1b, kalrn1, mfn2, pdia5, psmd13, rcor1, satb1, waspla, wasplb and wdr66, resulted in no observable phenotype. Depletion of brd3a, kalrn2 and kif1b resulted in a severe reduction in the number of primitive erythrocytes. All the embryos are positioned anterior up and dorsal to the back. (TIFF) [file pgen.1004450.s014.tiff]

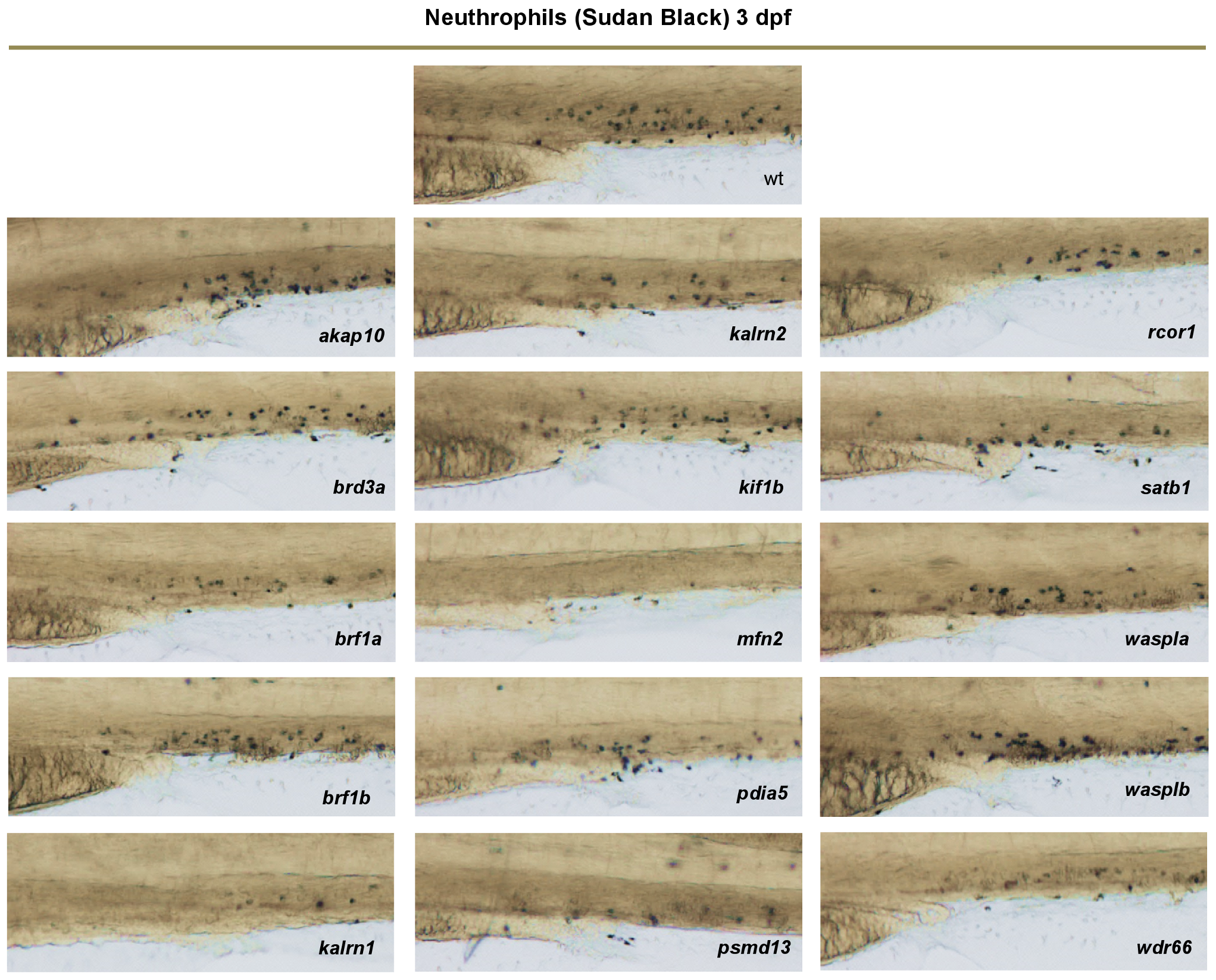

Supplement: Figure S15 — Individual neutrophils may be detected with Sudan Black staining. The number of neutrophils in MO-injected embryos (nembryos = 15) in CHT was counted at 3 dpf. Knock-down of akap10, brd3a, brf1b, kif1b, waspla or wasplb caused no phenotype. A mild decrease (20–50%) in the number of neutrophils was observed for brf1a, kalrn2, pdia5, rcor1, satb1 and wdr66 MO injected embryos compared to control and kalrn1, mfn2 and psmd13 MO injected embryos showed a severe (over 50%) depletion of neutrophils when compared to control. Representative images of the CHT region at 3 dpf are shown. All embryos are oriented with anterior to the left and dorsal to the top. (TIFF) [file pgen.1004450.s015.tiff]

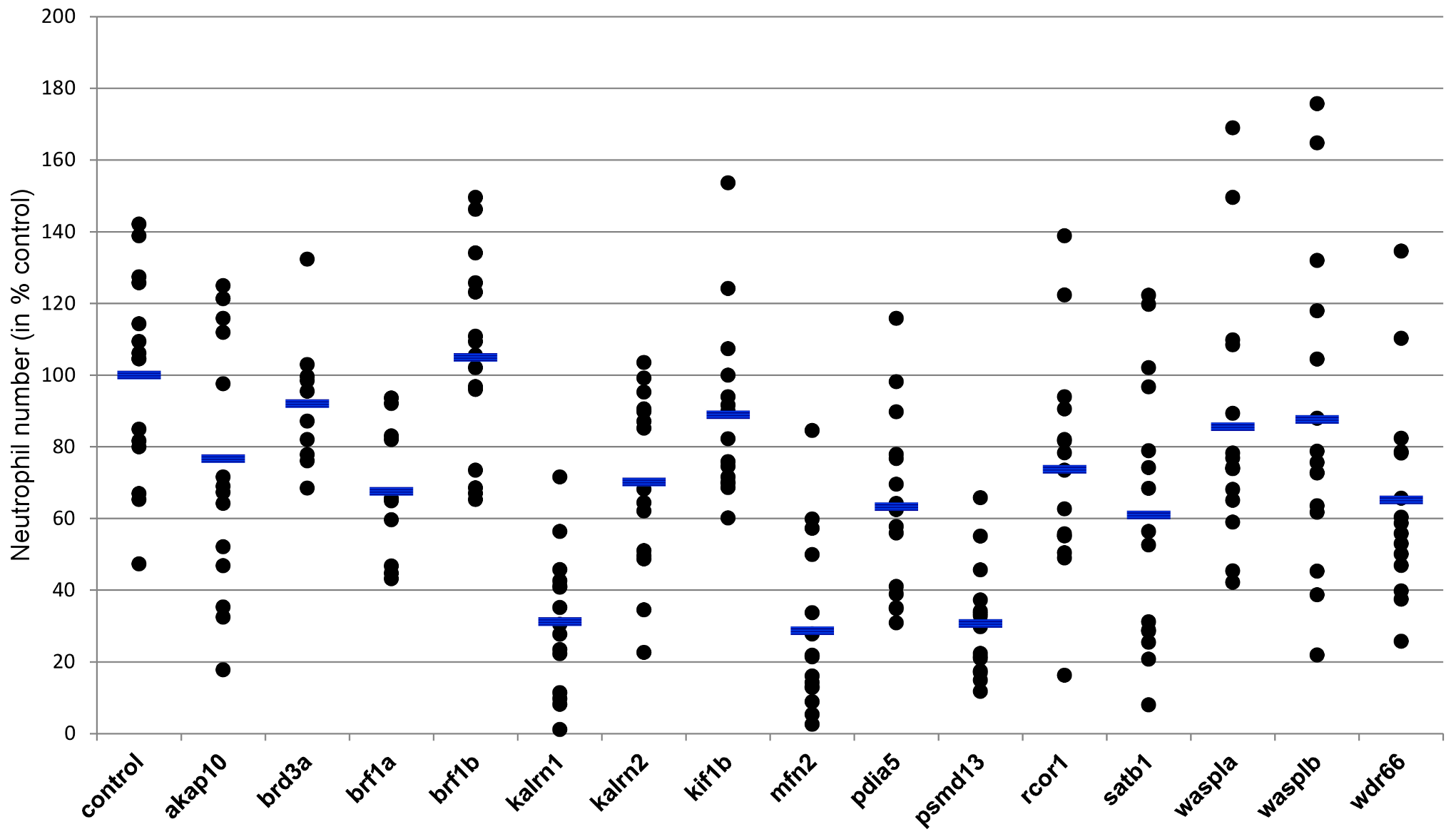

Supplement: Figure S16 — To identify neutrophils, Sudan Black staining was performed at 3 dpf. The number of neutrophils in CHT was counted in each group (nembryos = 15). One-tailed Student t test was performed. Significant decrease in the number of neutrophils was observed in brf1a (p = 0.019), kalrn1 (p = 3.1×10^−9^), kalrn2 (p = 0.004), mfn2 (p = 1.4×10^−6^), pdia5 (p = 0.004), psmd13 (p = 4.2×10^−11^), rcor1 (p = 0.012), satb1 (p = 0.011) and wdr66 (p = 9.2×10^−4^) MO-injected embryos. Each dot represents the number of neutrophils in an individual MO-injected embryo with respect to control. A blue horizontal line represents the mean value of the number of neutrophils for each group of embryos. (TIFF) [file pgen.1004450.s016.tiff]

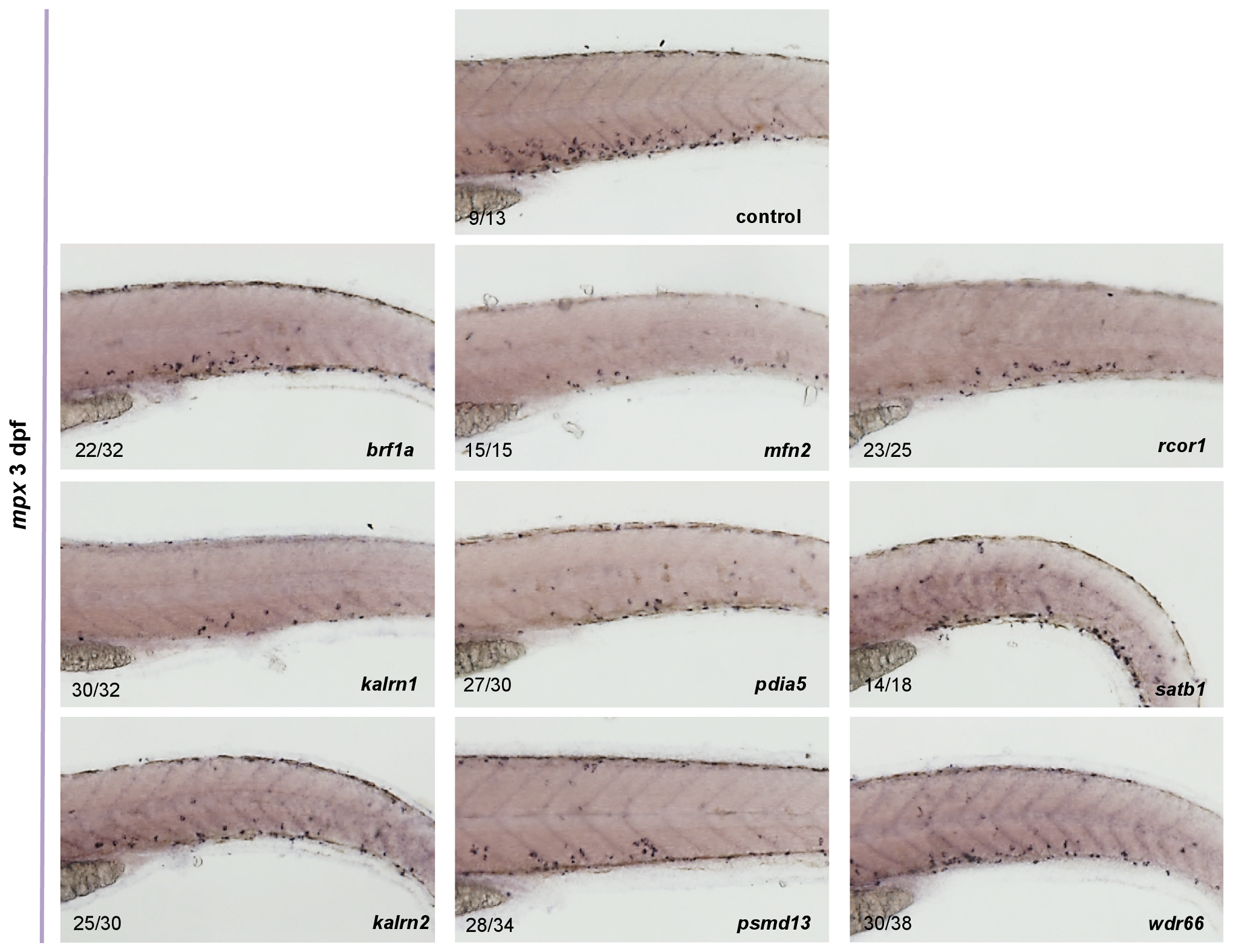

Supplement: Figure S17 — The expression of mpx in control as well as in brf1a, kalrn1, kalrn2, mfn2, pdia5, psmd13, rcor1, satb1 and wdr66 depleted embryos was assessed by in situ hybridization. For all genes tested the reduced number of mpx positive cells was observed when compared to the control. All embryos are oriented with anterior to the left and dorsal to the top. (TIFF) [file pgen.1004450.s017.tiff]

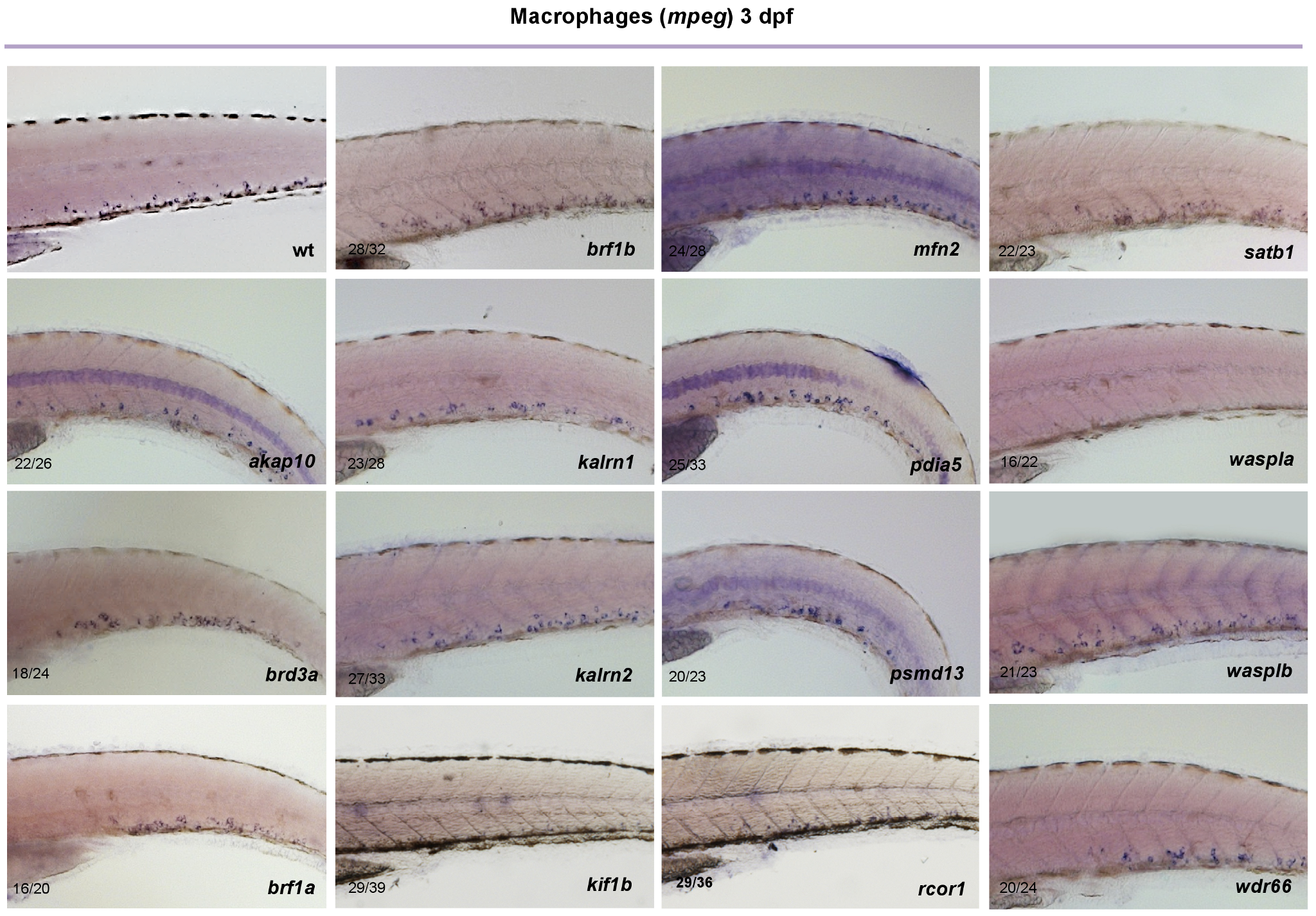

Supplement: Figure S18 — In order to detect macrophages, whole mount in situ hybridization was performed using the probe specific to mpeg1 at 3 dpf. For 13 out of 15 tested MOs (akap10, brd3a, brf1a, brf1b, kalrn1, kalrn2, mfn2, pdia5, psmd13, rcor1, satb1, wasplb and wdr66) there was no observable difference in the number of macrophages. However, knock down of kif1b and waspla resulted in a severe reduction in the number of macrophages in CHT at 3 dpf. All embryos are oriented with anterior to the left and dorsal to the top. (TIFF) [file pgen.1004450.s018.tiff]

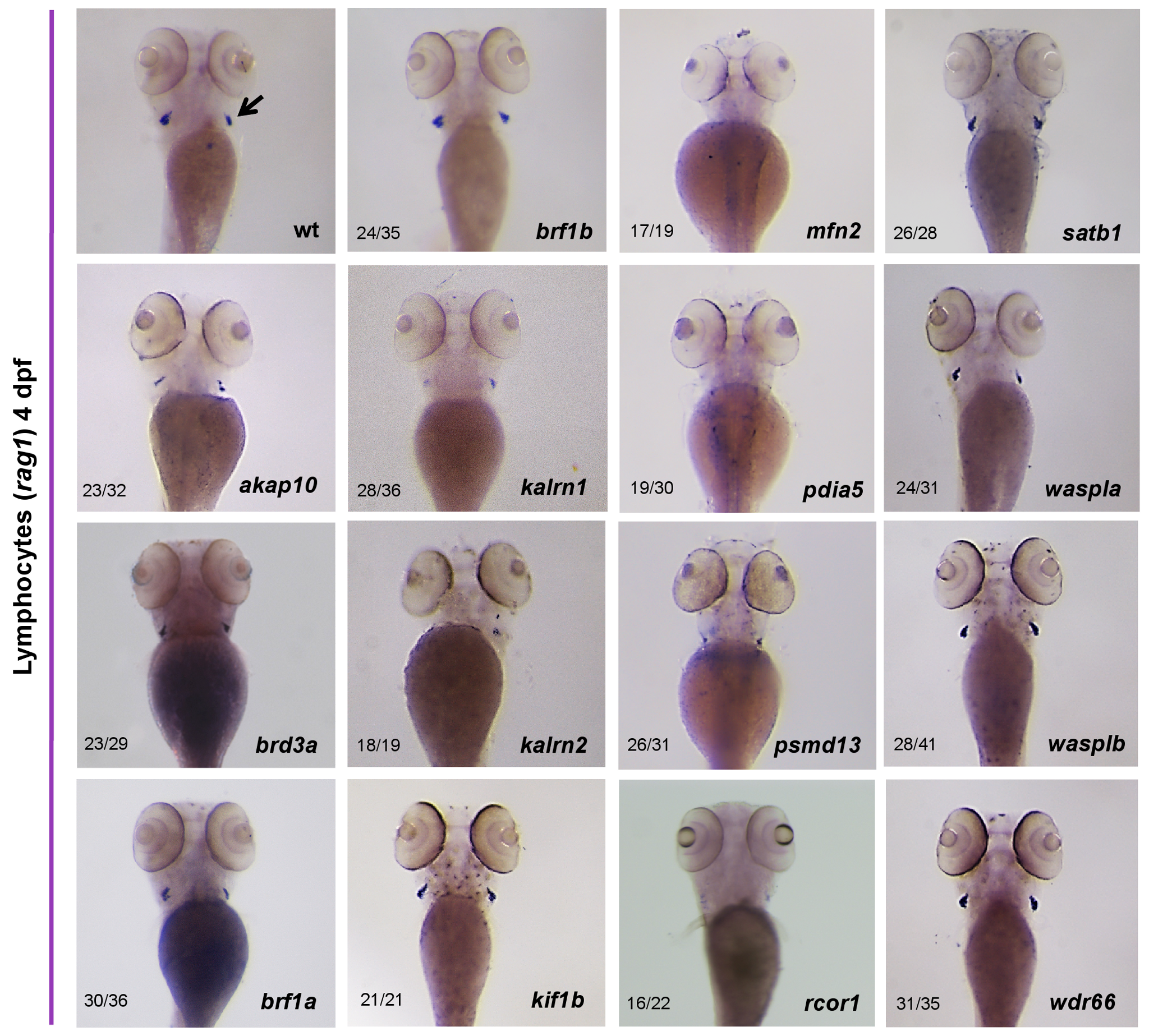

Supplement: Figure S19 — Differentiated thymic T-cells could be readily identified by rag1 expression when examined at 4 dpf. Whole mount in situ hybridization with a rag1 riboprobe revealed a severe decrease in the number of T lymphocytes in kalrn2, mfn2, pdia5 and psmd13 MO injected embryos in thymi (arrow) at 4 dpf. Knock down of akap10, kalrn1 and rcor1 caused a moderate decrease in T lymphocyte numbers. All the embryos are positioned anterior up and dorsal to the back. (TIFF) [file pgen.1004450.s019.tiff]

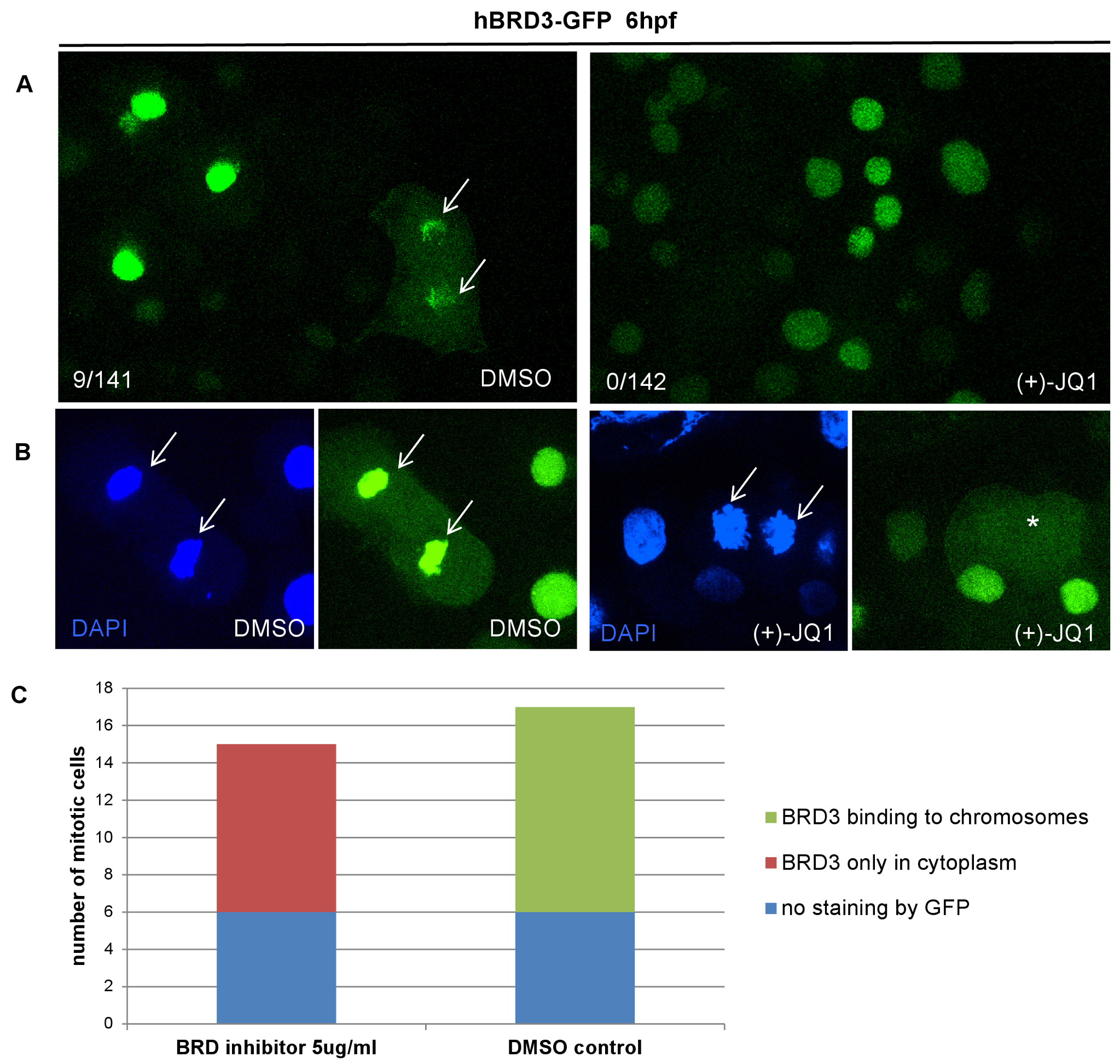

Supplement: Figure S20 — (+)-JQ1 is a highly specific inhibitor, which displaces BET proteins from chromatin by competitively binding to the acetyl-lysine recognition pocket of BET bromodomains. (A) Treatment of zebrafish embryos with (+)-JQ1 disrupted the chromatin occupancy of hBRD3-GFP as demonstrated by the absence of GFP-positive mitotic chromosomes in these embryos (0/142 GFP-positive cells) compared to 9/141 GFP-positive cells in the DMSO control group. (B–C) To further quantify the chromatin occupancy of hBRD3-GFP during mitosis, we selected ≥15 mitotic cells, as shown by DAPI staining, and counted how many of these cells were GFP positive in the presence of (+)-JQ1 or DMSO (control). Whereas in (+)-JQ1 treated embryos none of the DAPI positive mitotic chromosomes were GFP positive, in DMSO treated embryos 11 cells were double DAPI/GFP positive. Asterisk depicts GFP in the cytoplasm and arrow shows mitotic chromosomes. (TIFF) [file pgen.1004450.s020.tiff]

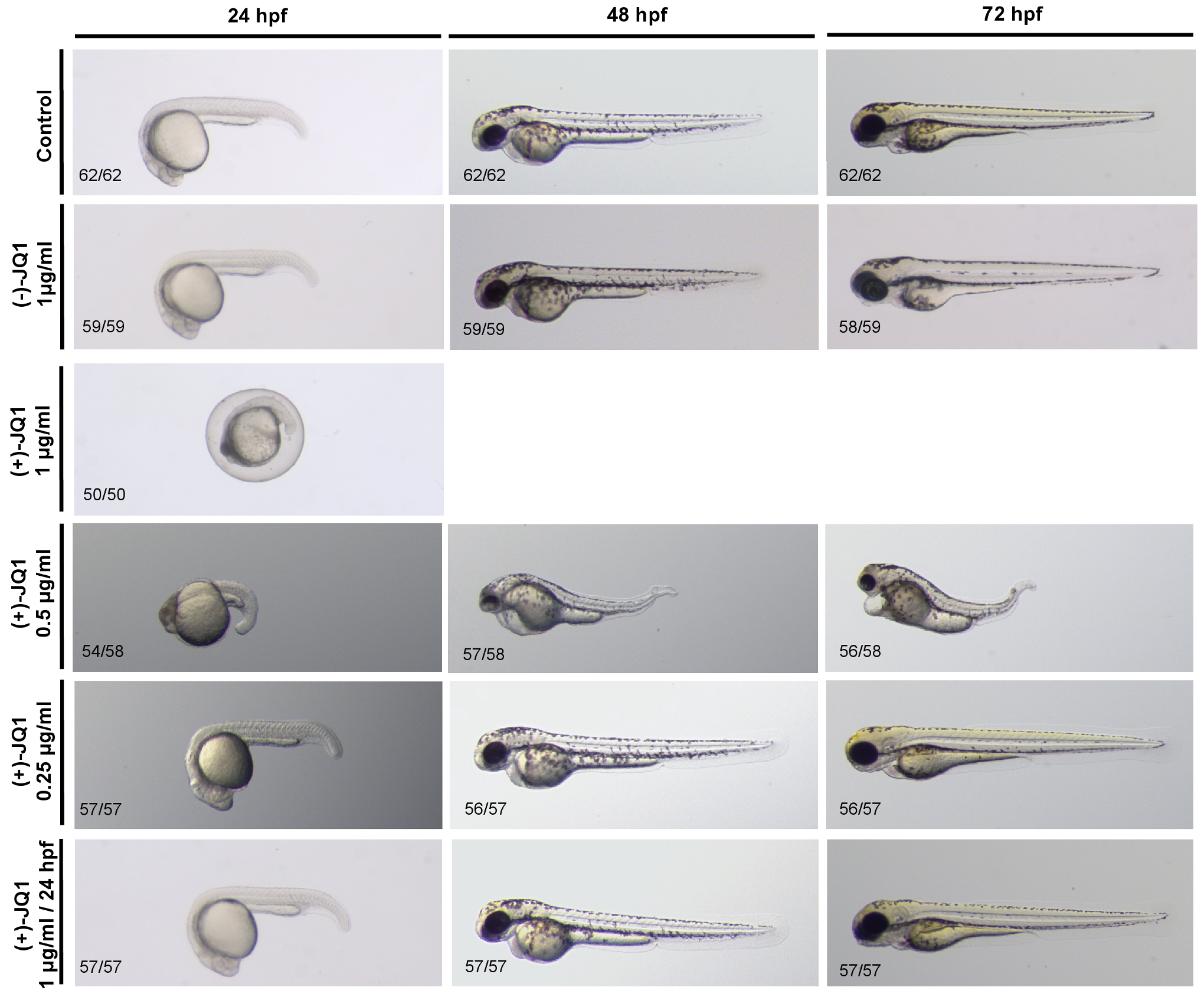

Supplement: Figure S21 — To assess the effect of the inhibitor on embryo development, dose response experiments were performed. Embryos treated with 1 µg/mL (+)-JQ1 from 6 hpf died by 24 hpf. The same concentration of the inactive enantiomer (−)-JQ1 did not affect the development of embryos. Incubation in lower concentration of (+)-JQ1 (0.5 µg/ml) led to aberrant development, i.e. tail malformation and heart edema. The lowest tested dose, 0.25 µg/ml, did not affect the development of the embryos. To avoid early embryonic lethality (+)-JQ1 inhibitor was added at 24 hpf. The embryos exhibited overall normal development even at the higher concentration (1 µg/ml) of (+)-JQ1. Representative images of embryo morphology are shown, taken at 24 hpf, 48 hpf and 72 hpf. All the embryos are positioned with anterior to the left and dorsal to the top. (TIFF) [file pgen.1004450.s021.tiff]

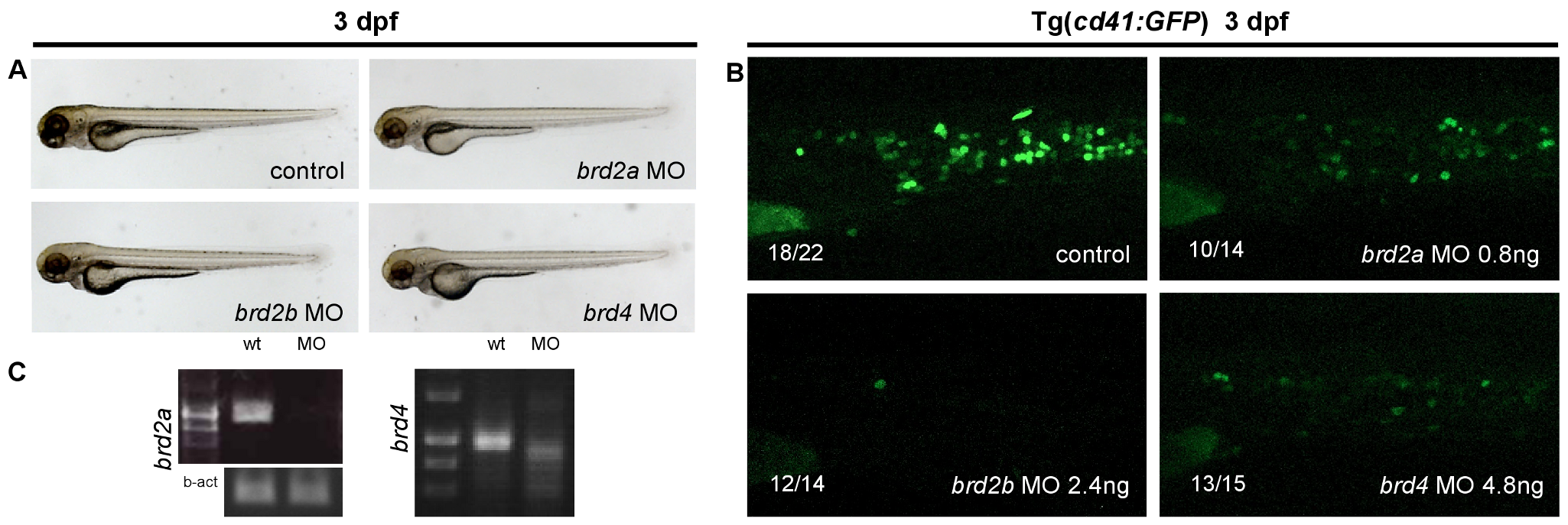

Supplement: Figure S22 — To assess the role of other BET family members in thrombopoiesis MO knock down of brd2a, brd2b and brd4 was performed. A) Although morphologically normal, embryos injected with brd2a, brd2b or brd4 MOs had a severe decrease in the number of thrombocytes at 3 dpf (B). Representative pictures of CHT are shown. C) For splice-blocking MOs the effect of the MOs was confirmed by RT-PCR. All the embryos are positioned with anterior to the left and dorsal to the top. (TIFF) [file pgen.1004450.s022.tiff]

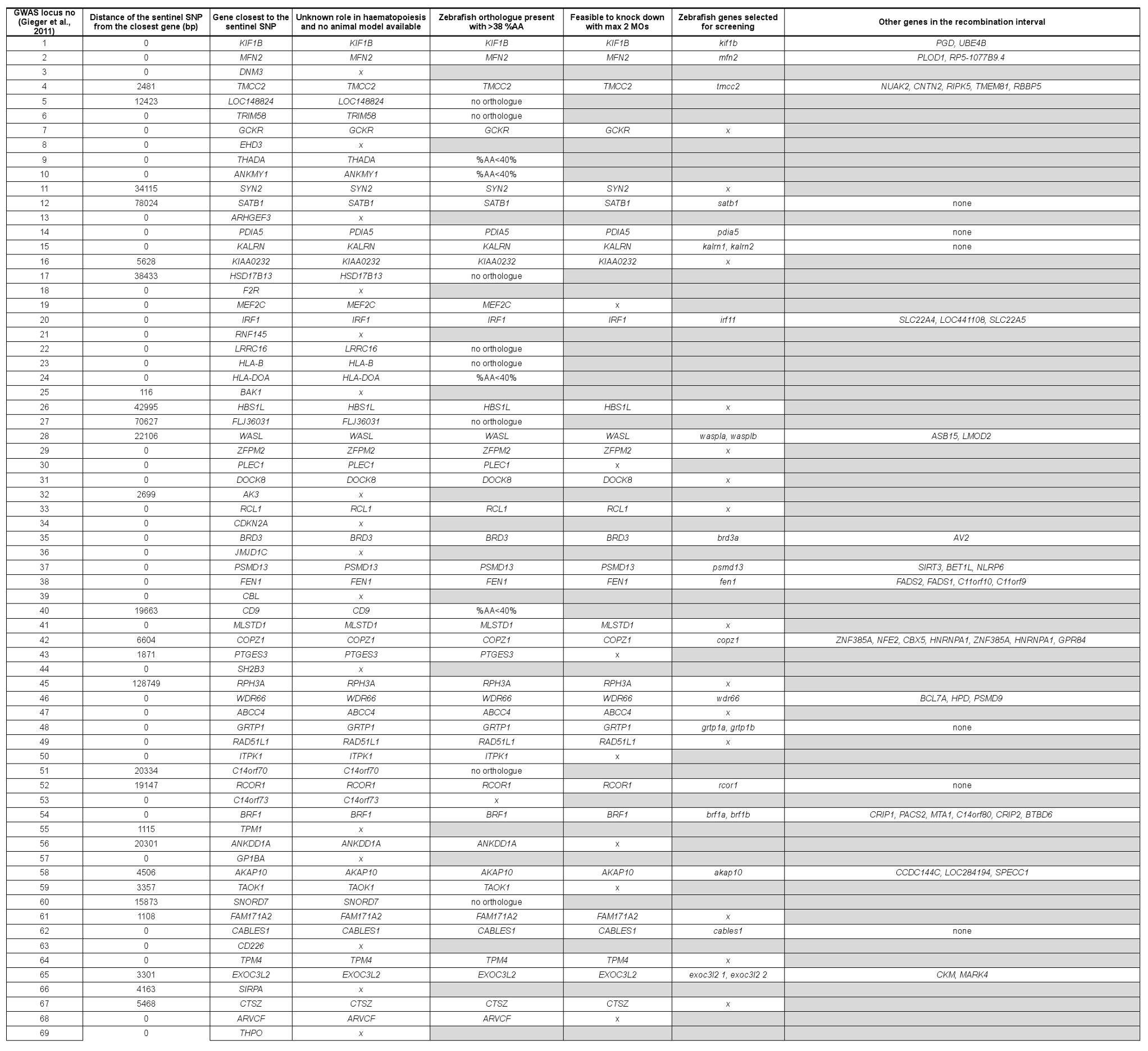

Supplement: Table S1 — Selection of the genes for in vivo functional screening. (TIFF) [file pgen.1004450.s023.tiff]

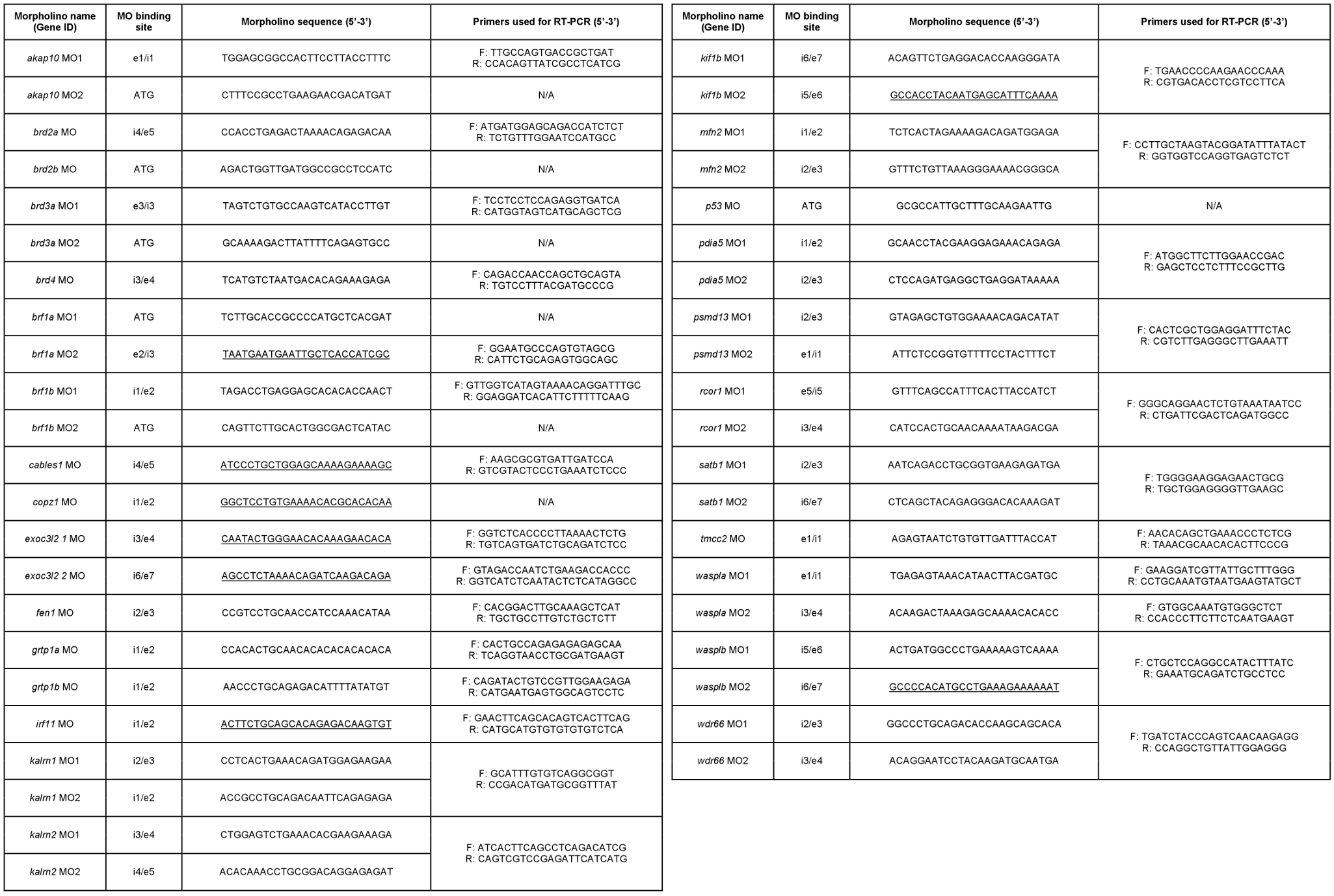

Supplement: Table S2 — Sequences of morpholinos and primers used in the study. Morpholinos with no effect on the splicing of their target transcript are underlined. (TIFF) [file pgen.1004450.s024.tiff]

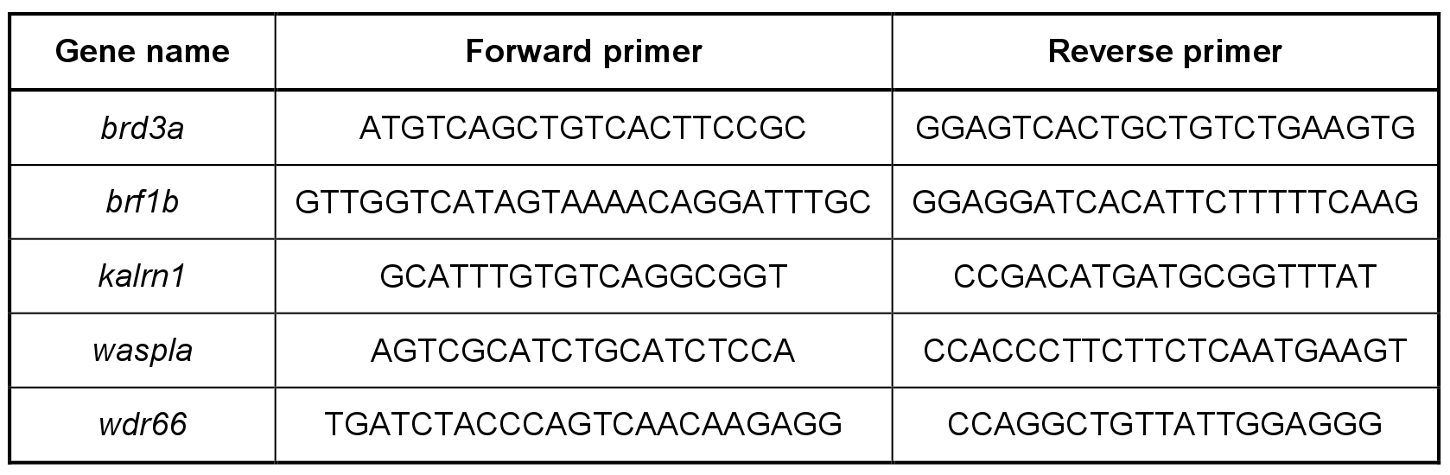

Supplement: Table S3 — Primers used for PCR amplification of candidate genes for probe synthesis. (TIFF) [file pgen.1004450.s025.tiff]
